# Lipoprotein(a) in Japanese Patients With Cardiovascular Disease: A Systematic Review

**DOI:** 10.1016/j.jacasi.2025.08.017

**Published:** 2025-10-07

**Authors:** Hiroshi Yoshida, Michel Kroes, Yoko Sakai, Yuri Takahashi, Yosuke Yamanaka, Bruce Crawford, Junya Ako

**Affiliations:** a Jikei University Kashiwa Hospital, Chiba, Japan; b Novartis Pharma, Basel, Switzerland; c Vista Health, Tokyo, Japan; d Novartis Pharma, Tokyo, Japan; e Kitasato University School of Medicine, Kanagawa, Japan

**Keywords:** cardiovascular disease, lipoprotein(a), secondary prevention

## Abstract

Lipoprotein(a) [Lp(a)] is recognized as an independent risk factor for cardiovascular disease (CVD), but its characterization within the Japanese population remains unexplored. This systematic literature review synthesizes evidence on the association between Lp(a) levels and CVD in Japanese patients. To ensure comparability, the review focused on studies using the widely used LATEX-based immunoassay method. Most studies categorized patients into “high” and “low” Lp(a) groups; this review concentrates on findings from the “high” groups to evaluate the impact of elevated Lp(a). Although definitions of “high” Lp(a) varied, a consistent association between elevated Lp(a) and increased cardiovascular risk has been observed, aligning with international findings. Variability across studies was noted, likely due to differences in study design, endpoints, and follow-up durations. Although no approved therapies specifically target elevated Lp(a), several randomized controlled trials are currently ongoing. Continued research is essential to better understand the clinical implications of elevated Lp(a) among Japanese individuals.

## Highlights

• Lp(a) is a known CV risk factor, but its clinical relevance in Japan is understudied.
• Elevated Lp(a) levels are associated with increased CV risk; standardized definitions remain critical.
• Future research should establish consensus definitions and CVD management strategies for elevated Lp(a) in Japan.

Lipoprotein(a) [Lp(a)] is a lipoprotein particle structurally similar to low-density lipoprotein, with the addition of apolipoprotein(a) linked via a disulfide bond to apolipoprotein B.^1^ Recent studies across diverse populations have increasingly recognized Lp(a) as an independent atherogenic factor associated with adverse cardiovascular (CV) outcomes such as myocardial infarction (MI), ischemic stroke, and aortic valve stenosis.^2^, ^3^, ^4^, ^5^ As such, elevated Lp(a) is increasingly acknowledged as a contributor to independent CV risk,^6^ which cannot be mitigated by conventional lipid-lowering therapies or lifestyle interventions, thereby requiring optimization of currently modifiable risk factors.^7^, ^8^, ^9^, ^10^ In particular, reducing Lp(a) levels is considered as a promising approach to mitigating CV risk among individuals with elevated Lp(a),^11^ and Lp(a) measurement is gaining clinical relevance, especially from a secondary prevention perspective.^6^

However, clinical handling of Lp(a) varies across regions. The 2022 Lp(a) consensus statement by the European Atherosclerosis Society proposed a pragmatic approach for clinical decision making, with Lp(a) cutoffs of <30 mg/dL (<75 nmol/L) for lower risk and >50 mg/dL (>125 nmol/L) for higher risk, while acknowledging a range in threshold values.^12^ Similarly, the American College of Cardiology and American Heart Association guidelines recognize ≥50 mg/dL (≥125 nmol/L) as a target for the primary prevention of cardiovascular disease (CVD).^13^ In contrast, although awareness of the importance of Lp(a) is growing in Japan, current domestic guidelines do not yet provide specific recommendations or judgement thresholds for Lp(a) measurement.^14^, ^15^, ^16^ In addition, it is known that Lp(a) values can vary depending on the assay kit used.^17^, ^18^, ^19^ Although it was demonstrated that elevated Lp(a) levels have been associated with increased CVD risk even if different assay kits are used,^20^ in Japan, where a standardized threshold has yet to be established, variation in assay kits may be one of the possible factors contributing to the challenges in interpreting the measurements in daily clinical practice.^21^

To enhance future clinical decision making and the development of national guidelines, it is essential to synthesize existing evidence on the relationship between Lp(a) and CVD in the Japanese population.

The present systematic literature review aims to synthesize the current evidence on Lp(a) and its association with CVD in Japanese patients. By focusing on this specific population, the review seeks to elucidate the relationship between Lp(a) levels and a variety of forms of CVD, including coronary artery disease (CAD), stroke, and peripheral artery disease (PAD). Because Lp(a) assay results can vary considerably depending on the measurement method, this review focuses on studies using the most commonly used LATEX-based immunoassay to ensure greater consistency and comparability in the synthesized evidence.

## Methods

### Search strategy

Relevant publications in English and Japanese indexed from January 1, 2003, to December 8, 2023, were identified through a search of the following computerized bibliographic databases: Embase, Medline, and Ichushi. Search string details are provided in Supplemental Tables 1-3. To broaden the search strategy, reference lists of included articles and 5 local medical associations’ online platforms were manually searched: the Japanese Circulation Society, the Japan Atherosclerosis Society, the Japanese College of Cardiology, the Japanese Association of Cardiovascular Intervention and Therapeutics, and the Japanese Society of Internal Medicine.

### Eligibility criteria

This review included study designs limited to systematic reviews, prospective or retrospective observational studies (evaluating ≥10 patients), interventional studies, modeling studies, and registries conducted in Japan. The original inclusion criteria for the patient population were broad, comprising adult patients with Lp(a) measurements in Japan. However, the criteria were refined to specifically target populations with CVD, including CAD, stroke, and PAD, thereby focusing the scope of the study. Predefined eligibility criteria were applied to the search results to identify all relevant references. All identified references were screened by 1 reviewer and checked by a second reviewer, with disagreements resolved by a third reviewer, using a 2-stage approach to reviewing the title and abstract and subsequently full texts for those references included in the title and abstract review. Studies were not selected based on methodologic quality, in order to include as many studies as possible.

Studies using LATEX-based assay kits (LATEX turbidimetric immunoassay kits or LATEX-enhanced turbidimetric immunoassay), more often used in Japan and more sensitive than turbidimetric immunoassay, were reported as the primary results. The overall results, regardless of the type of assay kit used, are presented in the Supplemental Material.

### Data extraction

Data on study details, populations, Lp(a) assessments, baseline and clinical characteristics, treatment patterns, and Lp(a)-associated burden were compiled into an extraction grid in Microsoft Excel.

### Statistical method

When reporting across studies, weighted means or medians were calculated by averaging the reported means or medians, weighted by the sample size of each study. The correlation between clinical burden and Lp(a) was analyzed with the use of the Pearson correlation coefficient.

## Results

### Study characteristics

**Figure 1:**
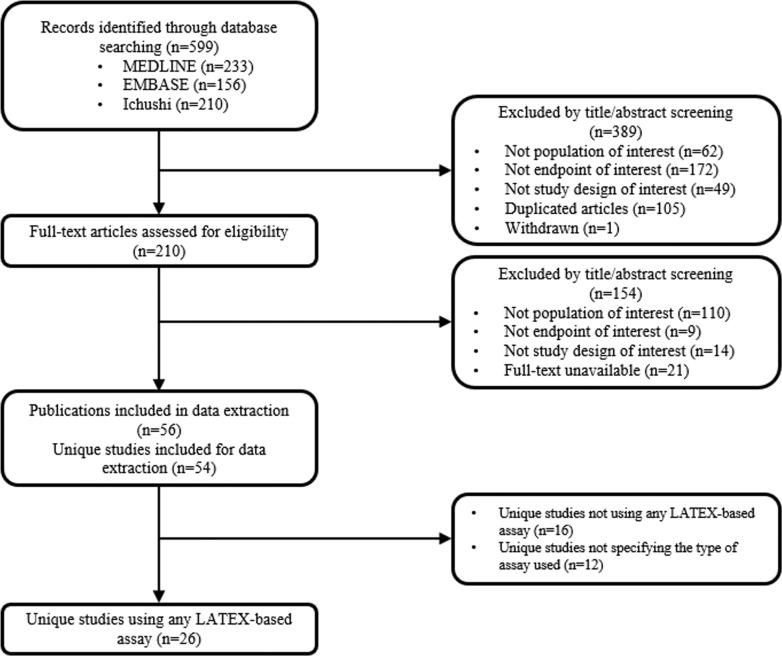
Flow Diagram for Selection of Studies for Inclusion The results of the search and selection process, from the number of records identified through the search to the number of studies included in the review, are presented.

A total of 599 citations were identified. After reviewing titles and abstracts, 210 full-text publications were obtained and assessed for relevance, of which 56 met the inclusion criteria (Figure 1). Data were extracted from a total of 56 publications. In addition, 2 studies each produced 2 different publications on the same patient population and design, as detailed in Supplemental Table 4. Twenty-six unique studies were identified that used LATEX-based assay kits to measure serum Lp(a). Sixteen studies were categorized as not using LATEX-based assay kits, while the remaining twelve studies did not specify the type of assay used (Supplemental Figure 1). Results from all studies, regardless of the type of assay, are presented in the Supplemental Material. Among the studies that reported using a LATEX-based assay, 11 (42.3%) studies were retrospective observational, 9 (34.6%) were prospective observational, 5 (19.2%) were randomized open-label, and 1 (3.8%) was a cross-sectional study. No modeling studies were identified (Table 1).

**Table 1:** Therapeutic Area, Study Design, and Included Patients in Each Study

Therapeutic Area	First Author (Year)	Population Characteristics	Study Design	Language
Acute CAD	Igarashi et al (2003)^35^	AMI patients with primary percutaneous transluminal coronary angioplasty	Prospective, observational	E
	Matsuda et al (2004)^23^	MI patients who underwent coronary angiography after onset and had a clearly discernible infarction-related artery	Retrospective, observational	E
	Mitsuda et al (2016)^33^	Patients with STEMI after PCI	Retrospective, observational	E
	Matsushita et al (2020)^39^	Patients with unstable angina pectoris, non- STEMI or STEMI	Prospective, randomized open-label	E
	Nakamura et al (2020)^63^	Patients with STEMI	Prospective, randomized open-label	E
	Kato et al (2022)^27^	Patients with ACS who underwent optical coherence tomography imaging of nonculprit plaques in the culprit’s vessels	Retrospective, observational	E
	Okubo et al (2023)^64^	Patients with ACS admitted to the hospital	Retrospective, observational	E
Chronic CAD	Hishikari et al (2020)^37^	Hemodialysis patients with stable angina pectoris who underwent PCI	Retrospective, observational	E
	Nakamura et al (2020)^63^	Patients with stable angina pectoris	Prospective, randomized open-label	E
CAD	Kajikawa et al (2006)^65^	Patients with angiographically determined coronary artery stenosis	Retrospective, observational	J
	Nozue et al (2014)^66^	Patients with angina pectoris	Prospective, randomized open-label	E
	Konishi et al (2015)^22^	Patients after PCI	Retrospective, observational	E
	Nozue et al (2016)^67^	Patients with angina pectoris	Prospective, randomized open-label	E
	Suwa et al (2017)^29^	Patients after PCI	Prospective, observational	E
	Shitara et al (2019)^30^	Patients after PCI	Prospective, observational	E
PAD	Hikita et al (2015)^68^	Patients with symptomatic PAD who underwent EVT	Retrospective, observational	E
	Hishikari et al (2017)^36^	Patients with PAD who underwent EVT	Retrospective, observational	E
	Yanaka et al (2021)^25^	Patients who underwent EVT for de novo femoropopliteal lesions	Retrospective, observational	E
	Tomoi et al (2022)^24^	Patients who underwent EVT for symptomatic PAD	Prospective, observational	E
CVD	Uchida et al (2003)^69^	ASCVD patients	Retrospective, observational	E
	Iwamoto et al (2004)^28^	ASCVD patients	Cross-sectional, observational	E
CKD with CAD	Konishi et al (2016)^32^	Patients with CKD who had been treated with PCI	Prospective, observational	E
DM with CAD	Murase et al (2008)^70^	DM patients with coronary heart disease	Prospective, observational	E
	Konishi et al (2016)^32^	DM patients who underwent PCI	Prospective, observational	E
	Takahashi et al (2020)^31^	DM patients who were treated with statin therapy at the time of PCI	Retrospective, observational	E
DM	Murase et al (2008)^70^	DM patients	Prospective, observational	E
Dyslipidemia	Nozue et al (2010)^71^	Dyslipidemia and history of CAD or DM	Prospective, observational	E

Several studies may be included in multiple categories as they include patients with different therapeutic areas.

ACS = acute coronary syndrome; AMI = acute myocardial infarction; ASCVD = atherosclerotic cardiovascular disease; CAD = coronary artery disease; CKD = chronic kidney disease; CVD = cardiovascular disease; DM = diabetes mellitus; E = English; EVT = endovascular therapy; J = Japanese; MI = myocardial infarction; PAD = peripheral arterial disease; PCI = percutaneous coronary intervention; STEMI = ST-segment elevation myocardial infarction.

### Population characteristics

The overall weighted mean age across the included studies was 68.3 years, with most studies including patients aged ≥60 years (Supplemental Table 5). Although the target population comprised patients with CVD, non-CVD patients were not excluded from the analysis if they were reported separately in the included studies, ensuring comprehensive reporting of all relevant results. In total, 9 diseases were included in the main analysis. These included CVD (n = 2), CAD (n = 6), acute CAD (n = 7), chronic CAD (n = 2), PAD (n = 4), chronic kidney disease (CKD) with CAD (n = 1), diabetes mellitus (DM) (n = 1), DM with CAD (n = 3), and dyslipidemia (n = 1) (Table 1). Several studies included patients with different types of diseases and therefore could be included in more than 1 category. In addition, men constituted the majority in most studies. The median percentage of current or past smokers was 41.6%. The proportions of smokers in each study ranged from 6% to 80%, with no notable differences between diseases. There were no consistent trends in lipid profiles, including total cholesterol, low-density lipoprotein cholesterol, high-density lipoprotein cholesterol, and triglycerides, when comparing across different diseases (Supplemental Table 5).

Hypertension and DM emerged as the most common comorbidities, reported in 25 studies (Supplemental Table 6). Excluding studies in which 100% of the subjects had either DM or CKD, hypertension was the most common comorbid condition in every study. The overall weighted mean prevalence of hypertension was 73.1%, with more than one-half of the subjects in all studies having hypertension. Studies with higher prevalences of hypertension tended to feature patients with a mean age exceeding the weighted mean.

### Mean or median Lp(a)

**Figure 2:**
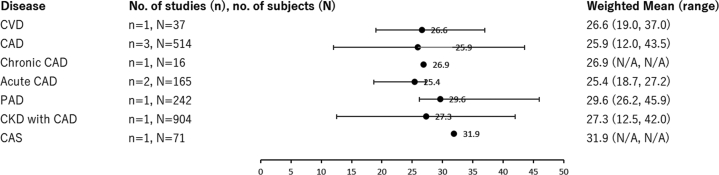
Weighted Averages and Ranges of Lp(a) Levels by Disease, mg/dL The weighted mean values indicated no notable differences in Lp(a) levels across different disease types. Weighted means were calculated by averaging the reported means, weighted by the sample size of each study. Several studies may be included in multiple categories as patients could have more than one condition. In several studies, Lp(a) levels were reported separately for the high Lp(a) and low Lp(a) groups. CAD = coronary artery disease; CAS = coronary artery stenosis; CKD = chronic kidney disease; CVD = cardiovascular disease; N/A = not applicable; PAD = peripheral arterial disease.

**Figure 3:**
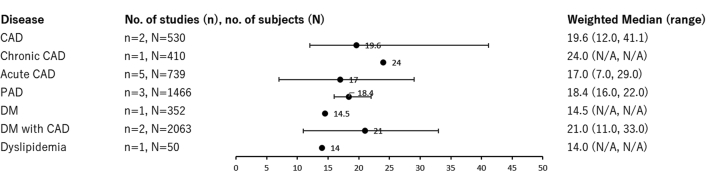
Weighted Averages of Medians and Ranges of Lp(a) Levels, mg/dL The weighted median values indicated no notable differences in Lp(a) levels across different disease types. Weighted medians were calculated by averaging the reported medians, weighted by the sample size of each study. Several studies may be included in multiple categories as patients could have more than one condition. CAD = coronary artery disease; DM = diabetes mellitus; N/A = not applicable; PAD = peripheral arterial disease.

Out of the 26 studies, mean or median Lp(a) values were reported in 25 studies. One study did not provide the median or mean Lp(a) levels of the included population; instead, the analysis was conducted by stratifying patients into high and low Lp(a) groups. All studies reported Lp(a) values in mg/dL. Regardless of the type of disease, the weighted mean of Lp(a) levels ranged from 25.4 to 31.9 mg/dL (Figure 2), and the weighted average of the median values ranged from 14.0 to 24.0 mg/dL (Figure 3). Both the weighted mean and weighted median values indicated no notable differences in Lp(a) levels across different disease types. However, when comparing CAD, chronic CAD, and acute CAD, Lp(a) levels tended to be higher in patients with chronic CAD compared with the other two groups. Similarly, when comparing DM and DM with CAD, Lp(a) levels were higher in patients with DM with CAD. It should be noted, however, that the number of studies and patients included that were available for each disease category was limited.

### Reported thresholds for high Lp(a)

**Figure 4:**
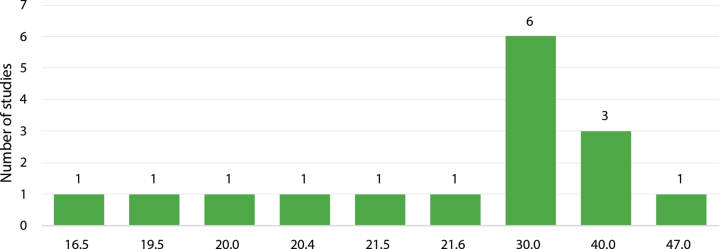
Threshold Studies Used to Define High Lp(a), mg/dL Although there were several thresholds used across studies, 30 mg/dL was used by the largest number of studies as the threshold for high Lp(a).

Seventeen of the 26 studies (65%) categorized participants into high Lp(a) and low Lp(a) groups within their analyses, with 6 of those studies using 30 mg/dL as the threshold to distinguish between high and low Lp(a) levels (Figure 4).^22^, ^23^, ^24^, ^25^, ^26^, ^27^ One of the studies defined patients with Lp(a) levels of ≥40 mg/dL as the “high Lp(a)” group, and those with Lp(a) levels <40 mg/dL were categorized as the “normal Lp(a)” group.^28^ The 17 studies that defined elevated Lp(a) levels used different approaches to define “high” Lp(a) levels. In 6 studies, the median Lp(a) value of the entire patient population was used as the threshold to define the high Lp(a) group, ranging from 16.5 mg/dL to 21.6 mg/dL.^29^^,^^30^, ^31^, ^32^, ^33^, ^34^ One study applied the Lp(a) value at the 75th percentile (47 mg/dL) of the population as the threshold,^35^ 6 studies established their thresholds without offering any rationale,^23^^,^^25^^,^^28^^,^^36^, ^37^, ^38^ and 4 studies referenced existing literature as their rationale for selecting a specific threshold.^22^^,^^24^^,^^27^^,^^39^ The cutoff values for the latter 4 studies ranged from 20 to 30 mg/dL, with 3 studies using 30 mg/dL. All 4 of those studies cited either the widespread use of this cutoff or the observed increase in CV risk as justification.

Those 4 studies were investigated to identify which research they referenced and the basis on which the cited studies defined the threshold (Supplemental Figure 3). Of the 4 studies, only Konishi et al (2015)^22^ referenced Abe and Noma (1992),^40^ which was conducted on a Japanese population; all the other studies cited research conducted outside of Japan. In addition, most of the cited studies referenced other papers to define the threshold.

### CV therapies used in patients with elevated Lp(a)

Thirteen studies reported medication use in patients categorized by high and low Lp(a), with aspirin, statins, angiotensin-converting enzyme inhibitors or angiotensin receptor blockers, beta-blockers, and calcium channel blockers being the most frequently reported (Table 2). At baseline, aspirin was the most frequently reported, although the usage rates varied widely, from 36.7% and 31.6% in high and low Lp(a) groups, respectively, with acute coronary syndrome (ACS),^27^ to more than 80% in other populations (Supplemental Table 7). No substantial differences in the use of other treatments were observed between the high Lp(a) and low Lp(a) groups.

**Table 2:** Treatment Usage by High Lp(a) or Low Lp(a) Group, %

		Aspirin	Statin	ACEI/ARB	BB	CCB
High Lp(a) group	Weighted average	94.4	72.3	54.4	49.7	39.4
	Range	36.7-100	46.9-100	29.5-100	18.4-85.0	19.2-55.6
Low Lp(a) group	Weighted average	94.0	69.4	55.7	49.4	41.7
	Range	31.6-98.9	42.0-100	42.1-88.0	27.9-86.0	20.8-51.9

ACEI = angiotensin-converting enzyme inhibitor; ARB = angiotensin receptor blocker; BB = beta-blocker; CCB = calcium channel blocker; Lp(a) = lipoprotein(a).

### Clinical burden

A total of 25 studies reported on the clinical burden associated with elevated Lp(a) levels. Major adverse cardiovascular events (MACEs) were the most frequently reported outcome (n = 7), followed by mortality outcomes, including cardiac, all-cause, and cancer-related deaths (n = 6). In addition, 4 studies reported CV events and ACS. MACE was defined as a composite of several major CV events, and the definition varied across studies (Table 3).

**Table 3:** Definition of MACE in Studies Reported

First Author (Year)	CV Event Included in MACE
Igarashi et al (2003)^35^	Cardiac death, nonfatal AMI, or recurrent angina
Mitsuda et al (2016)^33^	Cardiac death, nonfatal MI, coronary revascularization by myocardial ischemia in a de novo lesion, and ischemic stroke
Suwa et al (2017)^29^	Cardiac death and nonfatal acute coronary syndrome
Matsushita et al (2020)^39^	Death, MI, and any repeat revascularization
Hishikari et al (2020)^37^	Cardiac death, nonfatal MI, necessity of a new coronary revascularization procedure (coronary bypass surgery, repeat target lesion PCI, PCI for a new nontarget lesion)
Takahashi et al. (2020)^31^	Cardiac death, nonfatal MI, and nonfatal CI
Tomoi et al (2022)^24^	All-cause mortality, stroke, and MI

AMI = acute myocardial infarction; CI = cerebral infarction; MACE = major adverse cardiovascular events; MI = myocardial infarction; PCI = percutaneous coronary intervention.

**Figure 5:**
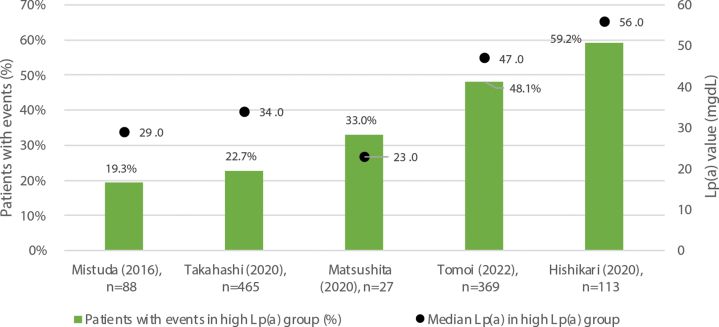
Median Lp(a) Levels and MACE in High Lp(a) Groups The bars represent the number of patients with events in the high Lp(a) group as reported in each study, and the black dots indicate the median Lp(a) level in the high Lp(a) group. A trend of higher MACE incidence rates was observed in groups with higher median Lp(a) levels (Pearson correlation coefficient = 0.85).

Among the 7 studies reporting MACE, 5 provided the median Lp(a) values and the proportion of patients who experienced MACE in the high Lp(a) group (Figure 5). A trend of higher MACE incidence rates was observed in groups with higher median Lp(a) levels (Pearson correlation coefficient = 0.85). Of these, Matsushita et al (2020) included patients with ACS, all of whom were treated with statins,^39^ in contrast to the variability in treatments reported in the other studies.^24^^,^^31^^,^^33^^,^^37^

**Figure 6:**
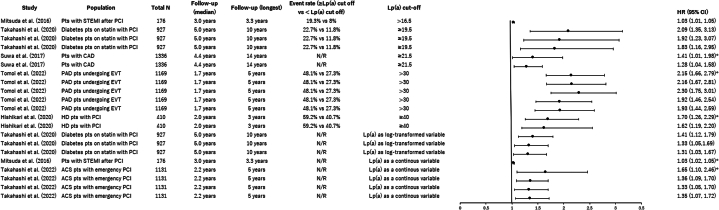
HRs for MACE, High Lp(a) vs Low Lp(a) Groups The black dots represent the HR values, and the horizontal lines indicate the 95% CIs. Reported HRs found significantly greater risk of major adverse cardiovascular events (MACE) in patients with high Lp(a) levels. ∗Univariate analysis; all of others are multivariable analyses. ACS = acute coronary syndrome; CAD = coronary artery disease; EVT = endovascular therapy; HD = hemodialysis; N/R = not reported; PAD = peripheral arterial disease; PCI = percutaneous coronary intervention; pts = patients; STEMI = ST-segment elevation myocardial infarction.

The reported analyses using Cox proportional hazard models consistently showed the risk of MACE to be significantly higher in subjects with elevated Lp(a) levels compared with those with lower Lp(a) levels (Figure 6). The HRs ranged from 1.03 (95% CI: 1.01-1.05) to 2.30 (95% CI: 1.76-3.01) across both univariate and multivariable analyses. Log-rank tests demonstrated that MACE occurred more frequently in patients with higher Lp(a) across 6 studies according to Kaplan-Meier (KM) analysis, regardless of the high Lp(a) thresholds (16.5 to 47 mg/dL) or the type of disease present in the patients (Table 4).^29^^,^^31^^,^^33^^,^^35^^,^^36^^,^^39^

**Table 4:** Log-Rank Test for MACE, High vs Low Lp(a) Groups

First Author (Year)	Population	N	Follow-Up, Median, y	Follow-Up, Longest, y	Lp(a) Cutoff, mg/dL	Event Rate, %, High vs Low Lp(a)	*P* Value
Mitsuda et al (2016)^33^	Patients with STEMI after PCI	176	NR	3	>16.5	NR	0.03
Takahashi et al (2020)^31^	DM patients with PCI	927	5.0	10	≥19.5	22.7 vs 11.8	<0.01
Matsushita et al (2020)^39^	ACS patients on statin	76	NR	5	>20	NR	0.02
Suwa et al (2017)^29^	Patients with CAD	1,336	4.4	NR	≥21.5	NR	<0.05
Hishikari et al (2020)^37^	Patients with stable angina pectoris with PCI	410	2.0	NR	≥40	59.2 vs 40.7	<0.01
Igarashi et al (2003)^35^	AMI patients with PTCA	127	2.9	NR	≥47	38.8 vs 9.4	<0.01

ACS = acute coronary syndrome; AMI = acute myocardial infarction; CAD = coronary artery disease; DM = diabetes mellitus; NR = not reported; PCI = percutaneous coronary intervention; PTCA = percutaneous transluminal coronary angioplasty; STEMI = ST-segment elevation myocardial infarction.

**Figure 7:**
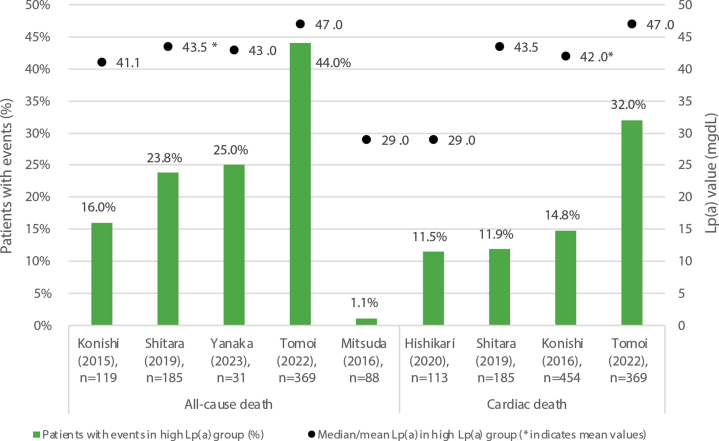
Median/Mean Lp(a) Levels and Mortality in High Lp(a) Groups The bars represent the number of patients with events in the high Lp(a) group as reported in each study, and the black dots indicate the median Lp(a) level in the high Lp(a) group. A trend similar to MACE outcomes was found for all-cause death, cardiac death, or both, with those with higher Lp(a) reporting higher mortality.

**Figure 8:**
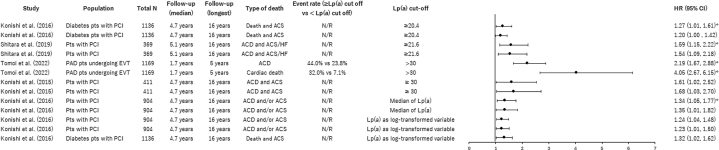
HRs for Mortality, High Lp(a) vs Low Lp(a) Groups The black dots represent the HR values, and the horizontal lines indicate the 95% CIs. Reported HRs found significantly greater risk of death in patients with high Lp(a) levels. ∗Univariate analysis; all of others are multivariable analyses. ACD = all-cause death; ACS = acute coronary syndrome; EVT = endovascular therapy; HF = heart failure; N/R = not reported; PAD = peripheral arterial disease; PCI = percutaneous coronary intervention; pts = patients.

Mortality outcomes, all-cause death, cardiac death, or both, followed a similar trend across the 6 studies (Figure 7). In 5 studies reporting HRs, high Lp(a) levels were significantly associated with an increased risk of mortality, including cardiac mortality, all-cause mortality, and other causes, with HRs ranging from 1.23 (95% CI: 1.01-1.50) to 4.05 (95% CI: 2.67-6.15) (Figure 8). In one study, significant results were not obtained when Lp(a) was analyzed as a categoric value divided into high and low groups (HR: 1.20; 95% CI: 1.00-1.42), whereas a log-transformed Lp(a) as a continuous variable was significant (HR: 1.32; 95% CI: 1.02-1.62).^32^

In contrast, the log-rank test for mortality comparing high vs low Lp(a) yielded mixed results (Table 5). Of the 4 studies that used Kaplan-Maier curves to estimate the unadjusted cumulative incidence of cardiac mortality and performed log-rank tests, only 1 demonstrated statistically significant differences between high and low Lp(a) groups.

**Table 5:** Log-Rank Test for Mortality, High vs Low Lp(a) Groups

First Author (Year)	Population	N	Follow-Up, Median, y	Follow-Up, Longest, y	Outcome	Lp(a) Cutoff, mg/dL	Event Rate, %, High vs Low Lp(a)	*P* Value
Konishi et al (2016)^32^	Diabetes patients with PCI	1,136	4.7	16.0	Death and ACS	≥20.4	NR	0.37
Hishikari et al (2020)^37^	HD patients with PCI	410	2.0	3.0	Cardiac mortality	≥40.0	11.5 vs 6.1	0.09
Igarashi et al (2003)^35^	AMI patients with primary PTCA	127	2.9	7.5	Cardiac mortality	≥47.0	0 vs 2.1	NS
Konishi et al (2016)^32^	CKD patients with PCI	904	4.7	16.0	Cardiac mortality	Median of Lp(a)	14.8 vs 8.4	<0.01

ACS = acute coronary syndrome; AMI = acute myocardial infarction; CKD = chronic kidney disease; HD = hemodialysis; NR = not reported; NS = not significant; PCI = percutaneous coronary intervention; PTCA = percutaneous transluminal coronary angioplasty.

Three studies individually reported the proportion of individuals who experienced CV events included in MACE^24^^,^^33^^,^^36^ (Supplemental Figure 8). The lowest MI incidence (2.3%) was observed in the study with the lowest median Lp(a), 29.0 mg/dL, and the highest (9.7%) in the study with the highest median Lp(a), 56.0 mg/dL.

**Figure 9:**
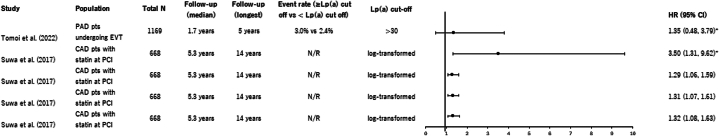
HRs for CV Event, High Lp(a) vs Low Lp(a) Groups The black dots represent the HR values, and the horizontal lines indicate the 95% CIs. Although using a categoric high/low Lp(a) did not find any significant differences in CV events, using Lp(a) as a continuous parameter (log-transformed) found significantly greater risk of CV events in patients with high Lp(a) levels. ∗Univariate analysis; all of others are multivariable analyses. Suwa et al (2017) reported the results of multivariate analysis (models 2-4 from top to bottom) with several different confounders (model 1: age and sex; model 2: model 1 + LDL-C, HDL-C, and TG; model 3: model 1 + variables with *P*<0.05 in univariable analysis, comprising diabetes, multivessel disease, and acute coronary syndrome). CAD = coronary artery disease; EVT = endovascular therapy; N/R = not reported; PAD = peripheral arterial disease; PCI = percutaneous coronary intervention; pts = patients.

Furthermore, 2 studies reported differing associations between Lp(a) levels and CV events as parts of MACE (Figure 9). One study found no significant association (HR: 1.35; 95% CI: 0.48-3.79), based solely on a univariate model.^24^ Another study treated Lp(a) as a natural log–transformed continuous variable, presenting results from both unadjusted and adjusted analyses.^29^ A notable discrepancy was observed between the univariate (HR: 3.50; 95% CI: 1.31-9.62) and multivariable (HRs ranging from 1.29 [95% CI: 1.06-1.59] to 1.32 [95% CI: 1.08-1.63]) analyses, highlighting the need for cautious interpretation of univariate results. Outcome definitions also varied, with one focusing on MI^24^ and another using the broader term “cardiac events.”^29^

The results for ACS also were mixed. Although ACS was reported in 4 studies, data on event incidence or the proportion of patients experiencing ACS were available in only 3 (Supplemental Figure 10).^30^^,^^33^^,^^34^ Although none of the studies estimated HRs, 2 of them used the log-rank test to examine differences in ACS incidence between the high and low Lp(a) groups and demonstrated statistically significant differences.^32^^,^^34^ Despite sharing data sources, these 2 studies analyzed distinct patient populations.

## Discussion

Elevated Lp(a) levels have been shown to contribute independently to CVD risk, regardless of the presence or number of conventional risk factors.^41^ The majority of studies examining elevated Lp(a) levels in the Japanese population reported a statistically significant relationship with clinical outcomes, particularly MACE, even when assays other than LATEX-based methods were used for Lp(a) measurement (Supplemental Figures 5 and 7). Results for mortality, CV events, and ACS were more variable, and several factors may explain this inconsistency. One key consideration is the variation in follow-up duration across studies,^42^ with median follow-up periods ranging from 1.7 years^24^ to 5.3 years^29^ and maximum follow-up periods ranging from 3 years^33^^,^^36^ to 16 years.^22^^,^^32^^,^^34^ Notably, studies with longer maximum follow-up periods (≥14 years) generally detected significant differences in event rates between high and low Lp(a) groups,^29^^,^^34^^,^^43^ suggesting that the impact of Lp(a) may become more apparent over time. This result is consistent with findings from previous studies conducted outside of Japan. Previous research has demonstrated that elevated Lp(a) levels are associated with an increased incidence of long-term adverse outcomes.^44^^,^^45^ Specifically, in patients with Lp(a) levels ≥125 nmol/L (58.1 mg/dL), each doubling of Lp(a) levels was associated with a 0.32% increase in atheroma volume over a 10-year follow-up period compared with those with Lp(a) levels <125 nmol/L.^44^ This suggests that elevated Lp(a) levels may have a long-term impact on the development of vulnerable coronary plaques, ultimately contributing to the occurrence of CVD. In addition, although Konishi et al (2016) did not observe a significant difference in cardiac death or ACS with the use of Kaplan-Meier analysis, significant association with elevated Lp(a) was revealed with the use of multivariable Cox models that accounted for demographic factors such as age and body mass index, along with clinical characteristics such as left ventricular ejection dysfunction and multivessel disease.^3^ These findings highlight the importance of sufficient follow-up duration and appropriate adjustment for confounding variables in clarifying the long-term clinical impact of elevated Lp(a), further reinforcing its role in CV risk assessment.

These trends are aligned with findings from studies conducted outside of Japan. A large prospective cohort study using data from the National Health and Nutrition Examination Survey, which monitors the health and nutrition of adults and children in the United States, demonstrated a significant association between elevated Lp(a) levels and the risk of all-cause mortality as well as CVD-related mortality, with a median follow-up period exceeding 20 years.^46^ Similarly, studies from Asia,^47^^,^^48^ Europe,^49^ and North America^50^ involving patients with CVD such as atherosclerotic CVD, familial hypercholesterolemia, MI, and PAD also reported a significant link between elevated Lp(a) levels and major CV events with different follow-up periods.^42^^,^^51^ Although differences in Lp(a) characteristics may exist across racial groups,^52^^,^^53^ these studies suggest that elevated Lp(a) levels are associated with outcomes such as MACE, MI, and coronary heart disease across various regions. Importantly, evidence from mendelian randomization and genome-wide association studies has established a causal role of Lp(a) in CV risk,^3^ further reinforcing its relevance as an independent contributor to atherosclerotic disease.

The assignment of high vs low Lp(a) remains a challenge in Japan owing to the lack of accepted standardization. Although many included studies in this review have referenced external sources to define high Lp(a) levels, some of them predominantly rely on research conducted outside of Japan, particularly European or American research, without demonstrating sufficient alignment regarding Lp(a)-related factors across different racial groups. Among the cited literature, 3 key papers were identified (highlighted in Supplemental Figure 3). Utermann (1989)^54^ focused on the genetics of Lp(a) and indicated that in Caucasians, a threshold of 30 mg/dL is associated with an increased risk of premature coronary heart disease among familial hypercholesterolemia patients. Kostner et al (1981) conducted a study on 76 male patients, aged 40-60 years, all of whom had suffered an MI at least 6 months earlier, demonstrating that using 30 mg/dL as a cutoff point, elevated Lp(a) represents an RR of 1.75 for MI in a normolipidemic population in Europe.^55^ These studies were influential, but they were published in and reflected the clinical context and population characteristics of the 1980s. Although the fundamental relationship between Lp(a) levels and CVD risk may have remained relatively consistent, it is imperative to consider the impact of evolving lifestyle factors,^56^^,^^57^ advances in Lp(a) measurement techniques,^58^ and the emergence of novel therapeutic options. Consequently, a re-evaluation of the Lp(a)–CVD risk association within the contemporary context is warranted.

Notably, the Japanese study cited in the literature reported no significant differences in Lp(a) concentrations between healthy Japanese individuals and Europeans.^40^ However, they also found significant differences when comparing healthy Japanese individuals with other Asian populations, specifically Chinese, Indian, and Malaysian groups. These differences have only been noted in healthy individuals, and it remains unclear whether theey exist for individuals at risk of CVD. Importantly, single-nucleotide polymorphisms (SNPs) linked to elevated Lp(a) in Western populations, such as rs10455872 and rs3798220, are rare in East and South Asians.^59^^,^^60^ In addition, the common SNPs differ between Japanese^61^ and Chinese populations,^62^ reflecting unique genetic characteristics. Given the potential for substantial racial variation, there is a need to advance discussions on the definition of high Lp(a) specifically for the Japanese population.

Currently, in Japan, the guidelines, such as the Japanese Circulation Society 2023 guideline on primary prevention of coronary artery disease, the Japan Atherosclerosis Society 2022 guidelines for prevention of atherosclerotic cardiovascular disease, and the Japanese Circulation Society 2018 guideline on diagnosis of chronic coronary heart diseases,^14^ have acknowledged the potential association between high Lp(a) and the risk of atherosclerotic disease.^14^, ^15^, ^16^ However, they do not define specific thresholds for high Lp(a). In addition, there are no guidelines for the management or treatment of high Lp(a) levels in Japanese patients, highlighting a gap in addressing high Lp(a) risk in this population.

The present review is, to the best of our knowledge, the first to examine and synthesize the definitions of high Lp(a) and its association with CV events in Japanese patients with CVD. The findings, focused on Japanese CVD patients, are consistent with studies conducted outside of Japan, which also demonstrate an elevated risk of CV-related events among patients with high Lp(a) levels.

However, this review has several limitations. A key limitation is the inconsistency in the definition of high Lp(a) used across the included studies, which obscures the precise risk level associated with varying Lp(a) thresholds. In addition, because the focus was on patients with established CVD, the results are primarily relevant to secondary prevention. To address these limitations, studies were categorized based on their definitions of high Lp(a), patient populations, and outcomes. This enabled a clearer examination of the association between Lp(a) levels and CV events. The analysis included a variety of study types, such as retrospective, prospective, cross-sectional observational studies, and randomized controlled trials. A formal quality assessment by study type was not conducted, because this was considered impractical given the anticipated heterogeneity and the limited number of studies within each category. Nevertheless, aspects of study quality and risk of bias were taken into account during the interpretation of findings, for example, by considering sample sizes, the appropriateness of control groups, and potential methodologic limitations such as short follow-up periods or lack of adjustment for confounding factors. The heterogeneity in study characteristics posed challenges for direct comparison of findings. While the inclusion of diverse study types allowed for a broader perspective, it may also have increased the risk of confounding and bias. Future analyses should consider stratifying or comparing results by study design to address these potential limitations more systematically. Moreover, some studies reporting CV outcomes considered only the first occurrence of a clinical event in their analyses. This methodologic approach may underestimate the cumulative burden of Lp(a)-associated risk over time and should be acknowledged as a further limitation. Finally, this review focused on categorizing Lp(a) into high and low groups to examine its relationship to CV risk. Exploring the association as a continuous variable may provide a more detailed understanding of the relationship between Lp(a) and CV risk, highlighting the need for further research.

## Conclusions

This review demonstrates that elevated Lp(a) is a risk factor for CVD in the Japanese population, highlighting the importance of managing Lp(a), particularly in the context of secondary prevention (Central Illustration). Despite the absence of a consistent definition for high Lp(a), the reviewed studies reported an association between elevated Lp(a) and an increased risk of CV events. These findings underscore the need for further research and the development of a consensus in Japan to help guide physicians in interpreting and managing Lp(a) levels alongside other risk factors. Research on therapeutic interventions specifically targeting elevated Lp(a) remains limited. Some early-phase studies have shown promising results, whereas treatments not primarily designed to lower Lp(a) have produced more variable outcomes, contributing to some inconsistency in the overall evidence base. Although robust evidence supporting the use of effective treatments to reduce the risks associated with high Lp(a) is still lacking, multiple randomized controlled trials are currently underway to address that gap. Further investigation in the Japanese population is therefore warranted to better quantify the impact of elevated Lp(a) on CV events to support future clinical decisions.

**Central Illustration:**
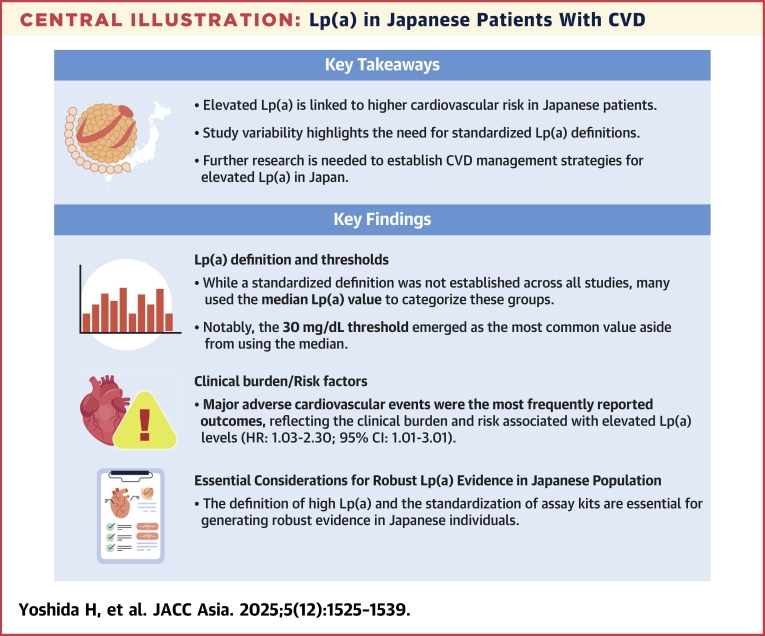
Lp(a) in Japanese Patients With CVD Evidence suggesting that high Lp(a) is a risk factor for CVD had not been comprehensively summarized in studies focusing on the Japanese population. This review demonstrated that individuals with high Lp(a) levels in Japan also have an increased risk of developing CVD, highlighting the importance of managing Lp(a), particularly in secondary-prevention settings. Moving forward, establishing a unified definition of what constitutes high Lp(a) in Japan will be crucial to better identify treatment targets.

## Funding Support and Author Disclosures

This systematic literature review and the preparation of the manuscript were funded by Novartis Pharma. Dr Yoshida has received honoraria for speaking activities from Kowa and Novartis and a manuscript fee from Denka and Kowa. Dr Kroes is an employee of Novartis Pharma. Mr Sakai and Mr Crawford are employees of Vista Health. Dr Takahashi and Mr Yamanaka are employees of Novartis Pharma. Dr Ako has received honoraria for speaking activities from Amgen, Astellas, AstraZeneca, Bayer, Novartis, Novo Nordisk, Mochida, Pfizer.
