# Supplementary material for: Lipoprotein(a) in Japanese Patients With Cardiovascular Disease: A Systematic Review
Source: JACC Asia. 2025 Oct 7;5(12):1525–39. doi: 10.1016/j.jacasi.2025.08.017 (PMC12794006; doi:10.1016/j.jacasi.2025.08.017)
Supplement: Supplementary Material [file mmc1.docx]

Supplemental Material

Supplemental Table 1. Embase search strategy (searched on 08 December 2023)

| **Parameter** | **Search terms** | **Hits** |
| --- | --- | --- |
| **Lp(a)** | (lipoprotein:ab,ti AND (‘lipoprotein(a)’ OR ‘Lp(a)’ OR ‘lipoprotein a’)) OR ‘lipoprotein(a)’/syn OR ‘Lp(a)’:ab,ti OR ‘lipoprotein(a)’:ab,ti OR ‘lipoprotein (a)’:ab,ti | 17,046 |
| **Epidemiology** | epidemiol* OR inciden* OR prevalen* OR trend* | 5,613,957 |
| **Screening** | screen* OR examin* OR detect* OR measur* OR value* OR evaluat* | 18,083,223 |
| **Disease management** | ‘standard treatment’:ab,ti OR ‘standard of care’:ab,ti OR combination:ab,ti OR agent*:ab,ti OR drug*:ab,ti OR treatment*:ab,ti OR therap*:ab,ti or regimen*:ab,ti OR pattern*:ab,ti OR algorithm:ab,ti OR registr* | 14,832,213 |
| **Burden** | ‘daly’:ab,ti OR ‘disability adjusted life year’ OR ‘qol’:ab,ti OR ‘quality of life’:ab,ti OR ‘quality-of-life’ OR ‘patient reported outcomes’:ab,ti OR ‘quality adjusted life year’ OR ‘qaly’:ab,ti OR (burden:ab,ti AND (‘clinical burden’ OR ‘humanistic burden’ OR ‘economic burden’ OR ‘caregiver burden’ OR ‘burden of illness’)) OR ‘cost*’:ab,ti OR ‘hospitali*’:ab,ti OR (‘emergency’ NEXT/2 (‘department’ OR ‘room’)) OR ‘utilit*’:ab,ti OR ‘comorbidity’:ab,ti OR ‘morbidity’:ab,ti OR ‘mortality’:ab,ti OR ‘death’:ab,ti OR ‘survival’:ab,ti OR ‘die’:ab,ti OR ‘adverse event’:ab,ti OR safety:ab,ti OR ‘side effect*’:ab,ti OR outpatient:ab,ti OR HCRU:ab,ti OR ‘healthcare resource utilization’:ab,ti OR risk*:ab,ti OR (‘long-term’:ab,ti AND outcome*:ab,ti) OR correlat*:ab,ti | 12,415,945 |
| **Filters and combination** | ‘editorial’/it OR ‘note’/it OR ‘commentary’/it | 1,737,469 |
|  | japanese(people) or Japanese | 1,002,163 |
|  | [english]/lim or [japanese]/lim | 38,363,043 |
|  | ([animal cell]/lim OR [animal experiment]/lim OR [animal model]/lim OR [animal tissue]/lim) NOT 'human'/de | 3,712,670 |
|  | [2003-2023]/py | 27,511,004 |
|  | #1 AND (#2 OR #3 OR #4 OR #5) AND #7 AND #8 AND #10 NOT #6 NOT #9 | 156 |

ab, ti: Finds designated syntax in the title and abstract

syn: Searchs the exploded term as well as all the synonyms listed

*: Truncation unlimited for the designated syntax

it: Filters articles by publication type

people: Filters articles by ethnic group

lim: Restricts searches based on publication type, language and availability of abstract, and database source

de: Filters articles by medical index term

py: Filters articles by publication year range

Supplemental Table 2. MEDLINE search strategy (searched on 08 December 2023 via Pubmed)

| **Parameter** | **Search terms** | **Hits** |
| --- | --- | --- |
| **Lp(a)** | (lipoprotein[tiab] AND ("lipoprotein(a)" OR "Lp(a)" OR "lipoprotein a")) OR "lipoprotein(a)"[Mesh] OR "Lp(a)"[tiab] OR "lipoprotein(a)"[tiab] OR "lipoprotein (a)"[tiab] | 11,161 |
| **Epidemiology** | epidemiol* OR inciden* OR prevalen* OR trend* | 4,708,660 |
| **Screening** | screen* OR examin* OR detect* OR measur* OR value* OR evaluat* | 13,595,435 |
| **Disease management** | "standard treatment"[tiab] OR "standard of care"[tiab] OR combination[tiab] OR agent*[tiab] OR drug*[tiab] OR treatment*[tiab] OR therap*[tiab] or regimen*[tiab] OR pattern*[tiab] OR algorithm[tiab] OR registr* | 11,228,789 |
| **Burden** | “daly”[tiab] OR “disability adjusted life year” OR “qol”[tiab] OR “quality of life”[tiab] OR “quality-of-life” OR "patient reported outcomes"[tiab] OR “quality adjusted life year” OR “qaly”[tiab] OR (burden[tiab] AND (“clinical burden” OR “humanistic burden” OR “economic burden” OR “caregiver burden” OR “burden of illness”)) OR “cost*”[tiab] OR “hospitali*”[tiab] OR (“emergency” NEXT/2 (“department” OR “room”)) OR “utilit*”[tiab] OR “comorbidity”[tiab] OR “morbidity”[tiab] OR “mortality”[tiab] OR “death”[tiab] OR “survival”[tiab] OR “die”[tiab] OR “adverse event”[tiab] OR safety[tiab] OR “side effect*”[tiab] OR outpatient[tiab] OR HCRU[tiab] OR “healthcare resource utilization”[tiab] OR risk*[tiab] OR (“long-term”[tiab] AND outcome*[tiab]) OR correlat*[tiab] | 8,882,899 |
| **Filters and combination** | editorial[publication type] OR news[publication type] OR comment[publication type] | 1,695,834 |
|  | Japan* | 1,957,445 |
|  | English[lang] or Japanese[lang] | 32,147,190 |
|  | Animals[Mesh] NOT (Humans[Mesh] AND Animals[Mesh]) | 5,174,651 |
|  | ("2003"[PDAT] : "2023"[PDAT]) | 21,334,533 |
|  | #1 AND (#2 OR #3 OR #4 OR #5) AND #7 AND #8 AND #10 NOT #6 NOT #9 | 233 |

MeSH: Medical Subject Headings, which is the National Library of Medicine's controlled vocabulary thesaurus used for indexing journal articles

*: Truncation unlimited for the designated syntax

TIAB: Title and abstract search field

NEXT/: The maximum number of words appearing between quoted search terms

PT: Filters articles with only the publication type of interest

LA: Filters articles with only the languages of interest

PDAT: Filters articles with only the published timeframe of interest

Supplemental Table 3. Ichushi search strategy (searched on 08 December 2023)

| **Parameter** | **Search terms** | **Hits** |
| --- | --- | --- |
| **Lp(a)** | "Lipoprotein(a)"/TH or "Lipoprotein(a)"/AL or "リポ蛋白(a)"/AL or "Lp(a)"/AL or "Lipoprotein a"/AL or “リポタンバクa”/AL or "リポ蛋白a"/AL | 4,726 |
| **Filters and combination** | AB=Y | 3,798,001 |
|  | 日本/TH or 日本/AL or Japan/AL or Japanese/AL | 9,999,249 |
|  | LA=日本語,英語 | 15,973,967 |
|  | (CK=ヒト) not (CK=小児) not (CK=青年期(13～18)) | 9,095,594 |
|  | (DT=2003/1/1:2023/12/08) | 7,514,029 |
|  | #1 and #2 and #3 and #4 and #5 and #6 | 210 |

TH: Consists of thesaurus indexed terms equivalent to PubMed "Mesh Term"

AL: Identifies all applicable search terms including partial matches

TA: Identifies all applicable search terms including partial matches, within the title and abstract

AB: Abstract availability

PT: Restricts publication type to designated categories

LA: Language restrictions

CK: Check tag limits searches from a specific perspective

DT: Publication year restrictions

Supplemental Table 4. Publications originating from the same study

| Title | Author | Year | Title | Author | Year |
| --- | --- | --- | --- | --- | --- |
| Lipoprotein(A) in familial hypercholesterolemia with proprotein convertase subtilisin/kexin type 9 (PCSK9) gain-of-function mutations | Tada et al. | 2016 | Lipoprotein (a) and the Risk of Chronic Kidney Disease in Hospitalized Japanese Patients | Tada et al. | 2020 |
| Relationship Between Lipoprotein(a) and Angiographic Severity of Femoropopliteal Lesions | Yanaka et al. | 2021 | Impact of lipoprotein(a) levels on primary patency after endovascular therapy for femoropopliteal lesions | Yanaka et al. | 2023 |

Supplemental Table 5. Patient baseline characteristics

|  |  | **Demographic characteristics** | | | | **Laboratory data** | | | |
| --- | --- | --- | --- | --- | --- | --- | --- | --- | --- |
| **Disease type** | **Study** | **Age (years)** | **Male (%)** | **BMI** | **Smoking (%)** | **TC** | **LDL-C** | **HDL-C** | **TG** |
| **CVD** | Uchida (2003), n=37 | 61.6 | 75.7 | - | 73.0 | 177.1 | 118.8 | 39.4 | 64.0 |
|  | Iwamoto (2004), n=208 | - | 47.6 | - | 26.0 | - | - | - | - |
| **CAD** | Kajikawa (2006), n=44 | 68.6 | 73.1 | - | 51.2 | 194.5 | 124.7 | 51.3 | 139.3 |
|  | Nozue (2014), n=119 | 66.4 | 83.2 | 24.4 | - | 205.0 | 131.5 | 46.2 | 130.8 |
|  | Konishi (2015), n=411 | 67.6 | 80.4 | 23.4 | 16.5 | - | 81.2 | 52.8 | 90.6 |
|  | Nozue (2016), n=101 | 67.0 | 83.0 | 24.3 | 23.0 | 202.0 | 129.0 | 47.0 | 114.0 |
|  | Suwa (2017), n=1336 | 64.5 | 82.1 | 24.6 | 25.8 | - | 111.0 | 45.0 | 139.3 |
|  | Shitara (2019), n=369 | 65.3 | 86.7 | 24.0 | 27.7 | - | 114.1 | 43.0 | 124.1 |
| **Acute CAD** | Igarashi (2003), n=127 | 66.3 | 81.8 | - | 68.1 | 185.9 | - | 46.0 | - |
|  | Matsuda (2004), n=129 | 59.3 | 82.2 | 23.3 | 67.4 | 194.2 | - | 41.0 | 139.0 |
|  | Mitsuda (2016), n=176 | 65.4 | 74.8 | - | 41.6 | 176.3 | - | - | - |
|  | Matsushita (2020), n=76 | 64.4 | 77.3 | 24.2 | 51.3 | 214.0 | 142.0 | 48.1 | - |
|  | Nakamura (2020), n=36 | 63.2 | 73.2 | 24.2 | 70.0 | 191.0 | 131.3 | 47.5 | 127.0 |
|  | Kato (2022), n=185 | 70.0 | 82.7 | - | 10.8 | - | - | - | - |
|  | Okubo (2023), n=175 | 66.5 | 85.7 | 24.3 | 70.9 | 172.7 | 107.4 | 48.8 | 105.4 |
| **Chronic CAD** | Nakamura (2020), n=16 | 61.6 | 92.0 | 24.9 | 69.0 | 168.6 | 114.4 | 49.3 | 156.5 |
|  | Hishikari (2020), n=410 | 70.8 | 76.8 | - | - | 140.0 | 71.0 | 43.0 | 116.0 |
| **PAD** | Hikita (2015), n=242 | 72.3 | 76.9 | 22.1 | 50.9 | - | 84.2 | 44.1 | 138.2 |
|  | Hishikari (2017), n=189 | 72.0 | 84.7 | - | 38.6 | - | - | - | - |
|  | Yanaka (2021), n=108 | 74.0 | 69.0 | 22.6 | 43.0 | 171.0 | 95.0 | 50.0 | - |
|  | Tomoi (2022), n=1169 | 74.9 | 70.1 | 22.7 | 19.0 | 166.3 | 96.9 | 51.6 | - |
| **CKD with CAD** | Konishi (2016), n=904 | 70.0 | 80.1 | 24.1 | 18.5 | - | 110.7 | 43.4 | 131.9 |
| **DM** | Murase (2008), n=327 | 62.0 | 66.7 | - | 42.0 | 215.0 | - | 54.0 | 124.0 |
| **DM with CAD** | Murase (2008), n=25 | 65.0 | 72.0 | - | 80.0 | 221.0 | - | 46.0 | 117.0 |
|  | Konishi (2016), n=1136 | 65.3 | 83.6 | 24.5 | 23.1 | - | 108.8 | 42.6 | 141.5 |
|  | Takahashi (2020), n=927 | 66.5 | 81.0 | 24.9 | 25.4 | - | 102.0 | 43.5 | 142.8 |
| **Dyslipidemia** | Nozue (2010), n=50 | 70.0 | 44.0 | 24.0 | 6.0 | 229.0 | 151.0 | 61.0 | 162.0 |

Note: Several studies may be included in multiple categories since patients can have more than one condition.

Abbreviations: BMI: body mass index; CAD: coronary artery disease; CKD: chronic kidney disease; CVD: cardiovascular disease; DM: diabetes mellitus; HDL: high-density lipoprotein; LDL: low-density lipoprotein; PAD: peripheral arterial disease; TC: total cholesterol; TG; triglyceride

Supplemental Table 6. Patient baseline comorbid conditions

| **Disease type** | **Study** | **Hypertension (%)** | **Diabetes (%)** | **Dyslipidaemia (%)** | **Multivessel disease (%)** | **CKD (%)** |
| --- | --- | --- | --- | --- | --- | --- |
| **CVD** | Uchida (2003), n=37 | 50.5 | 35.0 | - | - | - |
|  | Iwamoto (2004), n=208 | 53.5 | 16.5 | 34.2 | - | - |
| **CAD** | Kajikawa (2006), n=44 | 65.9 | 39.0 | - | - | - |
|  | Nozue (2014), n=119 | 63.1 | 42.2 | - | - | - |
|  | Konishi (2015), n=411 | 69.6 | 43.7 | - | 57.3 | - |
|  | Nozue (2016), n=101 | 65.0 | 45.0 | - | - | - |
|  | Suwa (2017), n=1336 | 70.8 | 44.7 | - | 59.7 | 25.1 |
|  | Shitara (2019), n=369 | 67.4 | 45.1 | - | 62.3 | 32.3 |
| **Acute CAD** | Igarashi (2003), n=127 | 59.1 | 28.3 | 31.5 | 23.1 | - |
|  | Matsuda (2004), n=129 | 59.3 | 41.9 | - | - | - |
|  | Mitsuda (2016), n=176 | 63.1 | 39.9 | 77.3 | 39.2 | - |
|  | Matsushita (2020), n=76 | 50.0 | 38.3 | - | - | - |
|  | Nakamura (2020), n=36 | 69.4 | 33.5 | - | 63.8 | - |
|  | Kato (2022), n=185 | 65.4 | 38.9 | 58.4 | - | - |
|  | Okubo (2023), n=175 | 66.5 | 32.0 | 70.9 | - | 25.1 |
| **Chronic CAD** | Nakamura (2020), n=16 | 69.0 | 38.0 | - | 56.0 | - |
|  | Hishikari (2020), n=410 | 72.2 | 69.8 | 36.8 | - | - |
| **PAD** | Hikita (2015), n=242 | 81.0 | 57.0 | 38.9 | - | 72.7 |
|  | Hishikari (2017), n=189 | 80.4 | 34.4 | 77.8 | - | - |
|  | Yanaka (2021), n=108 | 86.0 | 54.0 | 75.0 | - | 45.0 |
|  | Tomoi (2022), n=1169 | 89.2 | 51.3 | 72.5 | - | - |
| **CKD with CAD** | Konishi (2016), n=904 | 76.5 | 41.0 | - | 58.1 | 100 |
| **DM** | Murase (2008), n=327 | 63.9 | 100 | - | - | - |
| **DM with CAD** | Murase (2008), n=25 | 72.0 | 100 | - | - | - |
|  | Konishi (2016), n=1136 | 71.5 | 100 | - | 59.6 | - |
|  | Takahashi (2020), n=927 | 75.9 | 100 | 85.1 | 63.5 | 25.0 |
| **Dyslipidemia** | Nozue (2010), n=50 | 70.0 | 42.0 | 6.0 | - | - |

Note: Several studies may be included in multiple categories since patients can have more than one condition.

Abbreviations: CAD: coronary artery disease; CKD: chronic kidney disease; CVD: cardiovascular disease; DM: diabetes mellitus; PAD: peripheral arterial disease

Supplemental Table 7. Studies that report the proportion of patients using aspirin in the high and low Lp(a) groups

| **Disease type** | **Study** | **% of users among high Lp(a)** | **% of users among low Lp(a)** |
| --- | --- | --- | --- |
| ACS | Kato et al. (2022) | 36.7 | 31.6 |
| ACS with statin | Matsushita et al. (2020) | 100 | 98 |
| Angina | Hishikari et al. (2020) | 96.5 | 92.6 |
| CAD | Suwa et al. (2017) | 95.6 | 95.8 |
| CKD with PCI | Konishi et al. (2016) | 95.2 | 96 |
| DM with CAD | Konishi et al. (2016) | 94.2 | 93.9 |
| DM with CAD | Takahashi et al. (2020) | 97.2 | 97.6 |
| PAD | Hishikari et al. (2017) | 90.7 | 92.6 |
| PAD | Yanaka et al. (2021) | 83 | 81 |
| PCI | Konishi et al. (2015) | 92.4 | 93.5 |
| PCI | Shitara et al. (2019) | 90.2 | 91.3 |
| STEMI | Mitsuda et al. (2016) | 97.7 | 98.9 |

Abbreviations: ACS: acute coronary syndrome; CAD: coronary artery disease; CKD: chronic kidney disease; DM: diabetes mellitus; PAD: peripheral arterial disease; PCI: percutaneous coronary intervention; STEMI: ST-segment elevation myocardial infarction

Supplemental Table 8. Definition of MACE in studies reported across studies utilizing all different types of Lp(a) measurement kits

| **Study** | **Items included in MACE** |
| --- | --- |
| Dai et al. (2023) | Cardiac death, nonfatal MI, and rehospitalisation for heart failure |
| Kimura et al. (2022) | Cardiac death, MI, ST, clinically driven TLR, and revascularisation for new lesions |
| Igarashi et al. (2011) | Cardiac death, MI and/or revascularization for new lesions |
| Takahashi et al. (2020) | Cardiovascular death, non-fatal MI, and non-fatal cerebral infarction |
| Matsushita et al. (2020) | Death, myocardial infarction, and any repeat revascularization |
| Igarashi et al. (2003) | Cardiac death, nonfatal AMI, or recurrent angina. |
| Tomoi et al. (2022) | All-cause mortality, stroke, and MI. |
| Sakata et al. (2022) | All-cause death, non-fatal CVD, transient ischemic attack, or nonfatal myocardial infarction |
| Mitsuda et al. (2016) | Cardiac death, nonfatal MI, coronary revascularization by myocardial ischemia in a de novo lesion, and ischemic stroke. |
| Hishikari et al. (2020) | Cardiac death, nonfatal MI, necessity of a new coronary revascularization procedure (coronary bypass surgery, repeat target lesion PCI, PCI for a new non-target lesion) |
| Takahashi et al. (2022) | All-cause death and MI |
| Suwa et al. (2017) | Cardiac death and non-fatal acute coronary syndrome |

Abbreviations: AMI: acute myocardial infarction; CI: cerebral infarction; CVD: cerebrovascular disease; MACE: Major adverse cardiovascular events; MI: myocardial infarction; PCI: percutaneous coronary intervention; ST: stent thrombosis; TLR: target lesion revascularization

Supplemental Table 9. Log-rank test for ACS (high vs. low Lp(a) groups)

| **Study** | **Population** | **Total N** | **Follow-up (median)** | **Follow-up (longest)** | **Lp(a) cut off** | **Event rate (≥Lp(a) cut off vs < Lp(a) cut off)** | **P-value** |
| --- | --- | --- | --- | --- | --- | --- | --- |
| **Konishi (2016)** | Diabetes patients with PCI | 1136 | 4.7 years | 16.0 years | ≥20.4 | NR | 0.03 |
| **Konishi (2016)** | CKD patients with PCI | 904 | 4.7 years | 16.0 years | Median of Lp(a) | 7.9% vs 4.2% | 0.01 |

Abbreviations: CKD: chronic kidney disease; NR: not reported; PCI: percutaneous coronary intervention

Supplemental Table 10. Association between Lp(a) and risk factors of CV events

| **Author (Year)** | **Patient population** | **Correlation between** | **n** | **Spearman / Pearson / Other** | **Regression coefficient** | **P value** |
| --- | --- | --- | --- | --- | --- | --- |
| Nozue et al. (2016) | Patients with angina pectoris | LDL-C | 101 | Univariate linear regression | 0.14 | 0.15 |
| Nozue et al. (2016) | Patients with angina pectoris | HDL-C | 101 | Univariate linear regression | 0.13 | 0.20 |
| Nozue et al. (2016) | Patients with angina pectoris | TC | 101 | Univariate linear regression | 0.20 | 0.05 |
| Nozue et al. (2016) | Patients with angina pectoris | TC | 101 | Multivariate linear regression | -0.24 | 0.24 |
| Nozue et al. (2016) | Patients with angina pectoris | TG | 101 | Univariate linear regression | 0.03 | 0.77 |
| Nozue et al. (2016) | Patients with angina pectoris | Age | 101 | Univariate linear regression | 0.13 | 0.20 |
| Nozue et al. (2016) | Patients with angina pectoris | Sex | 101 | Univariate linear regression | -0.03 | 0.77 |
| Nozue et al. (2016) | Patients with angina pectoris | BMI | 101 | Univariate linear regression | -0.18 | 0.08 |
| Nozue et al. (2016) | Patients with angina pectoris | BMI | 101 | Multivariate linear regression | -0.32 | <0.01 |
| Nozue et al. (2016) | Patients with angina pectoris | Smoking | 101 | Univariate linear regression | -0.19 | 0.06 |
| Nozue et al. (2016) | Patients with angina pectoris | Smoking | 101 | Multivariate linear regression | -0.27 | <0.01 |
| Nozue et al. (2016) | Patients with angina pectoris | Hypertension | 101 | Univariate linear regression | 0.06 | 0.55 |
| Nozue et al. (2016) | Patients with angina pectoris | Diabetes | 101 | Univariate linear regression | -0.11 | 0.29 |

Note: Only Nozue et al. (2016) showed no significant association between serum lipids and Lp(a) using a univariate linear regression.

Abbreviations: BMI: body mass index; HDL: high-density lipoprotein; LDL: low-density lipoprotein; TC: total cholesterol; TG; triglyceride


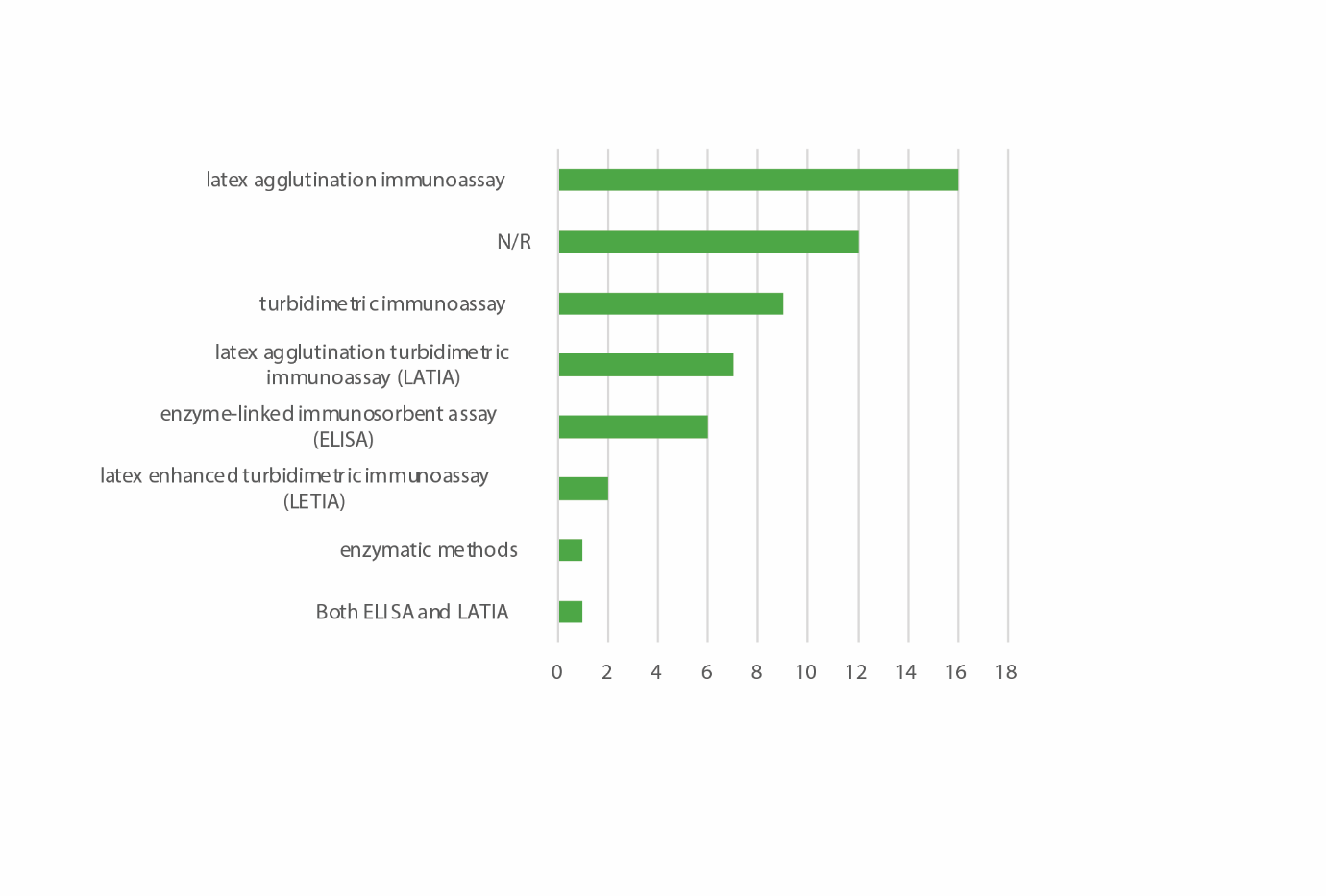


Supplemental Figure 1. Frequency of assay kit type used to measure Lp(a) levels

Twenty-six unique studies were identified that used latex-based assay kits to measure serum Lp(a). Sixteen studies were categorized as not using latex-based assay kits, while the remaining twelve studies did not specify the type of assay used.

Abbreviation: N/R: not reported


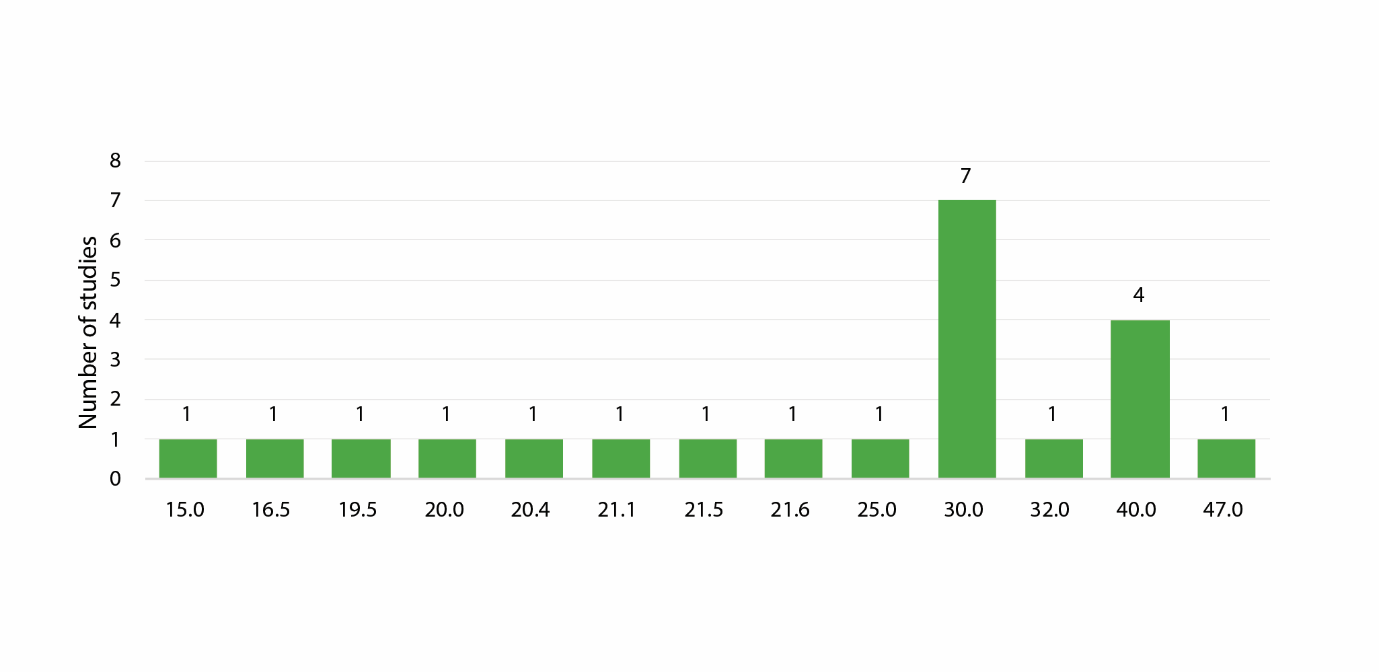


Supplemental Figure 2. Thresholds to define high Lp(a) across all Lp(a) measurement kits

Although there were several thresholds used across studies, 30 mg/dL was used by the largest number of studies as the threshold. Lp(a) measurement kit did not appear to influence the choice of high Lp(a) threshold.


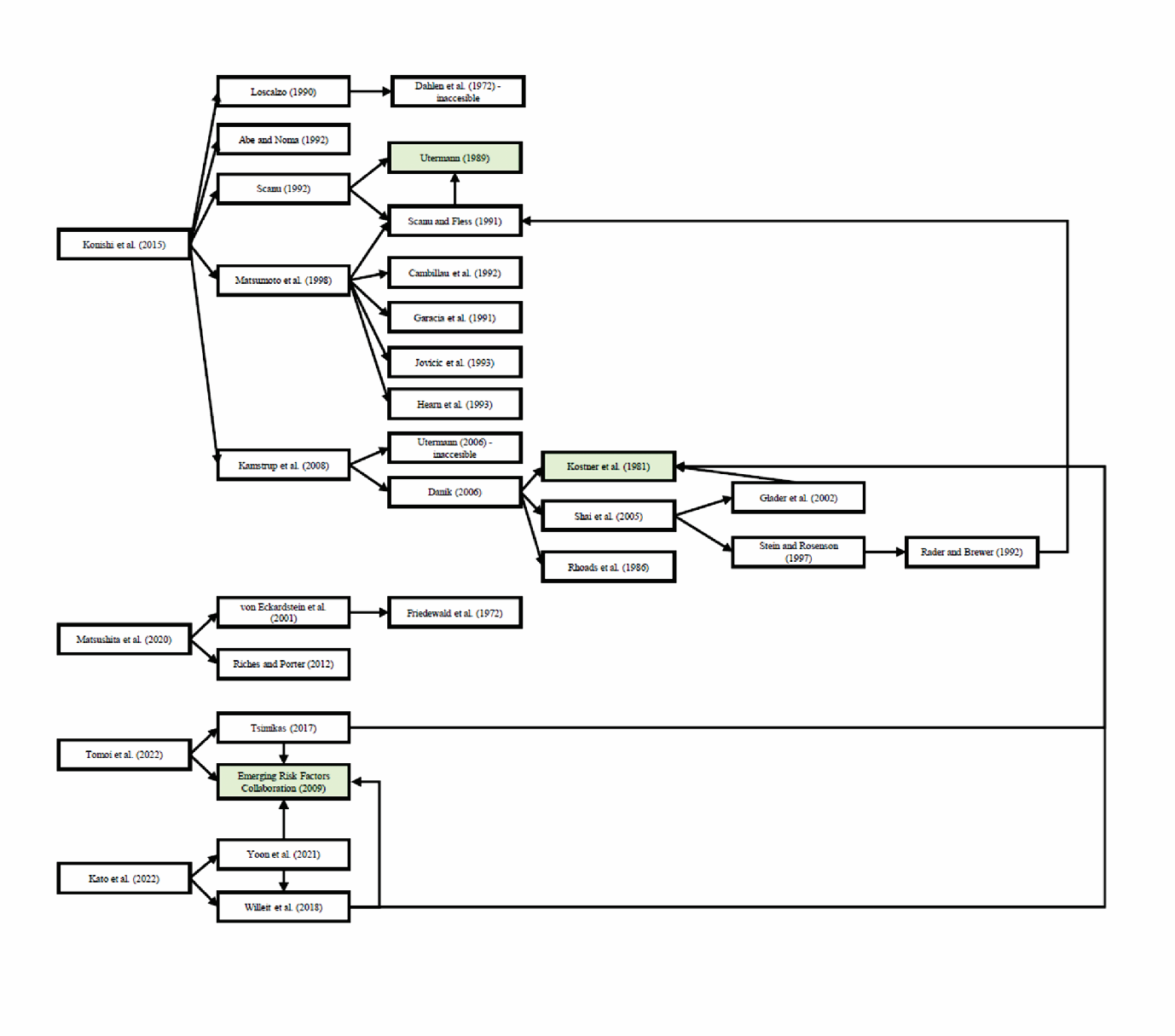


Supplemental Figure 3. Connections between studies referenced for threshold of elevated Lp(a)

Articles referencing thresholds used in other papers were traced back to their origin to identify how they were derived. Key articles are highlighted in green above.


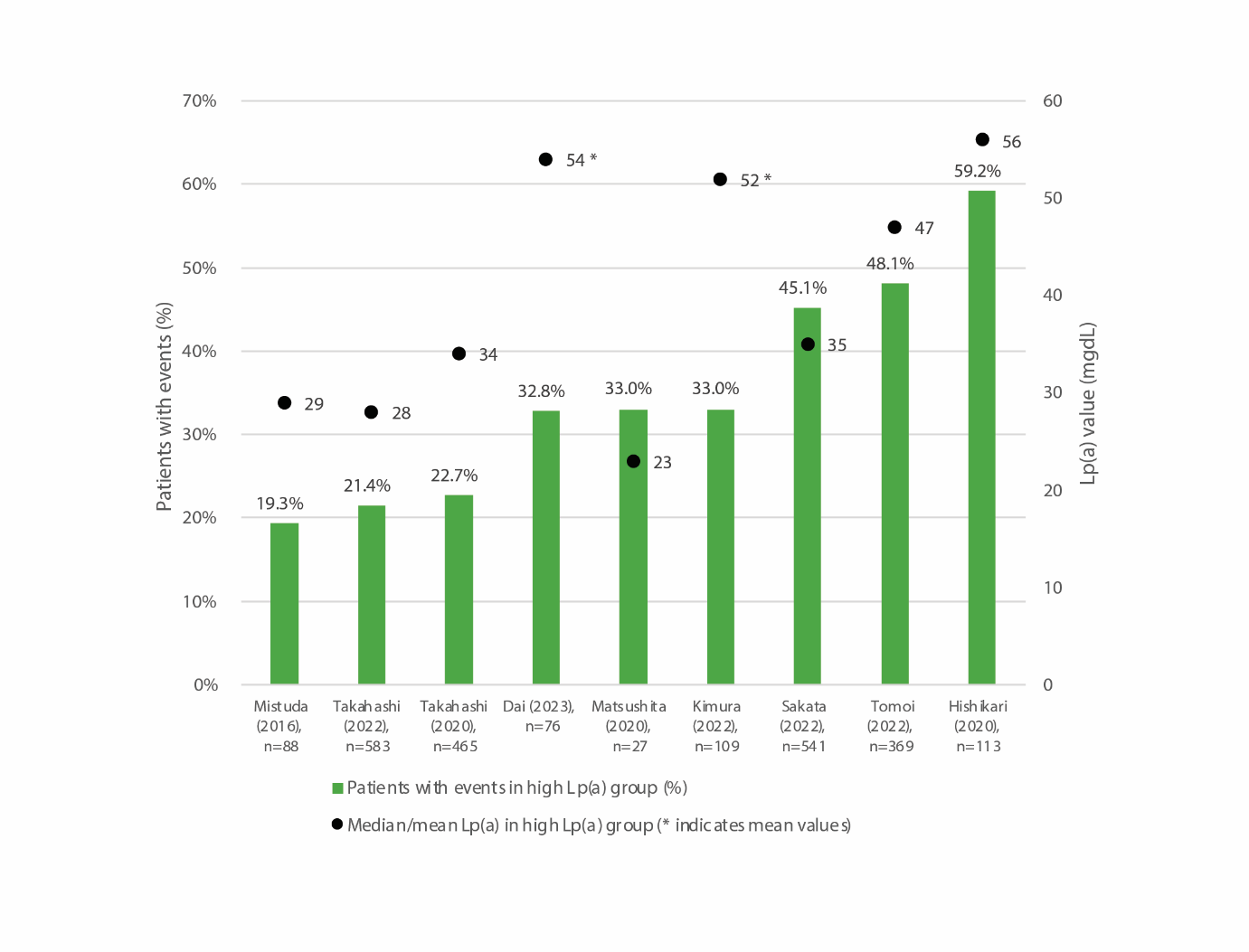


Supplemental Figure 4. Median Lp(a) levels and MACE utilizing all Lp(a) measurement kits

A trend of higher MACE incidence rates was observed in groups with higher median/mean Lp(a) levels.


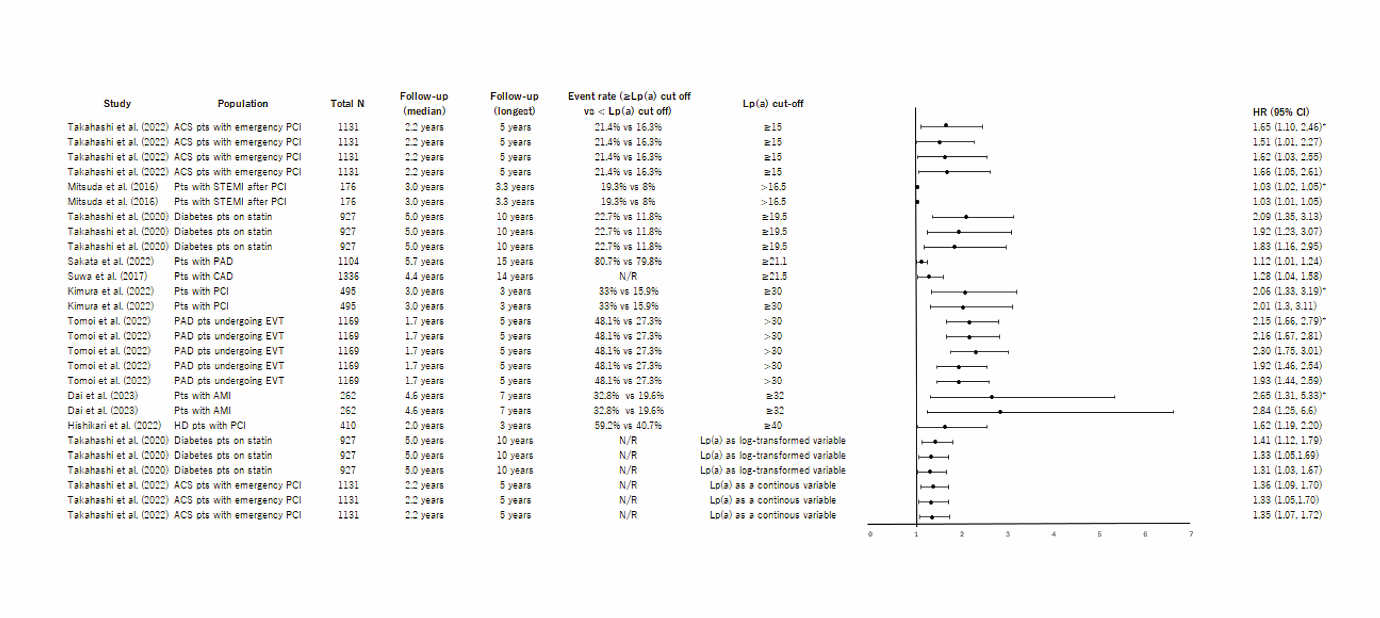


Supplemental Figure 5. Hazard ratios for MACE utilizing all Lp(a) measurement kits

The black dots represent the hazard ratios values, and the horizontal lines indicate the 95% confidence intervals. Reported hazard ratios found significantly greater risk of MACE in patients with high Lp(a) levels, regardless of Lp(a) measurement kit used. Asterisks (*) indicate univariate analysis, while all others are multivariate analysis

Abbreviations: ACS: acute coronary syndrome; AMI: acute myocardial infarction; CAD: coronary artery disease; EVT: endovascular therapy; HD: hemodialysis; N/R: not reported; PAD: peripheral arterial disease; PCI: percutaneous coronary intervention; Pts: patients; STEMI: ST-elevated myocardial infarction


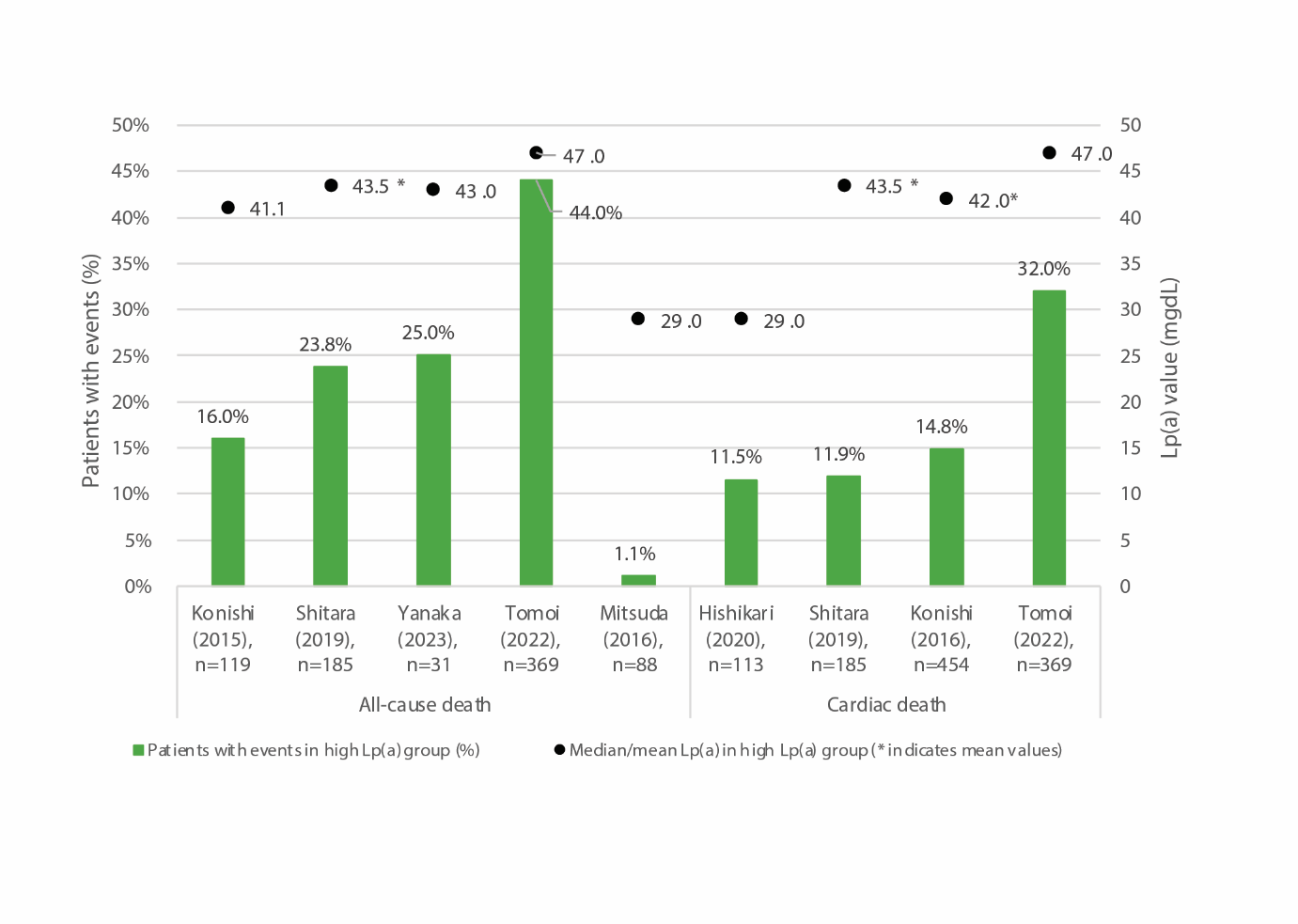
Supplemental Figure 6. Lp(a) levels and mortality utilizing all Lp(a) measurement kits

The studies reporting mortality in high Lp(a) utilized latex-based; therefore, the overall results remain unchanged.


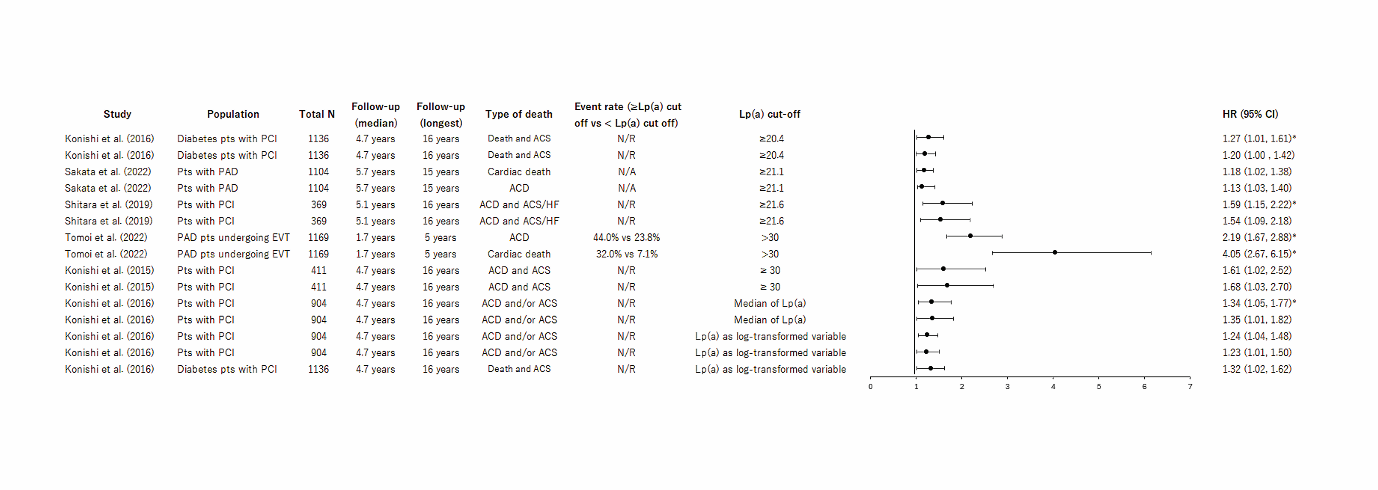


Supplemental Figure 7. Hazard ratios for mortality (high Lp(a) vs. low Lp(a) groups)

The black dots represent the hazard ratios values, and the horizontal lines indicate the 95% confidence intervals. Reported hazard ratios found significantly greater risk of death in patients with high Lp(a) levels, regardless of Lp(a) measurement kit used. Asterisks (*) indicate univariate analysis, while all others are multivariate analysis.

Abbreviations: ACD: all-cause death; ACS: acute coronary syndrome; EVT: endovascular therapy; HF: heart failure; N/R: not reported; PAD: peripheral arterial disease; PCI: percutaneous coronary intervention; Pts: patients


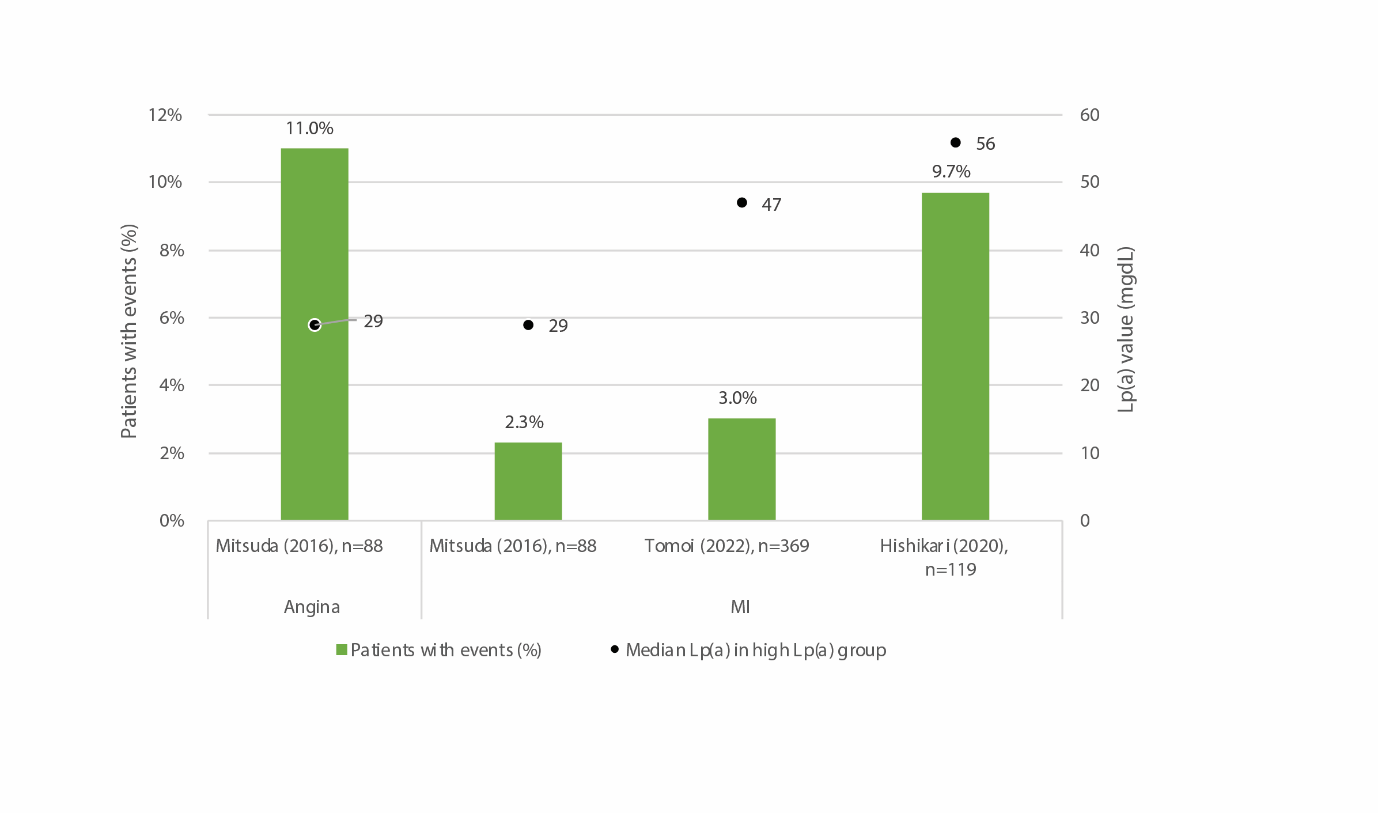


Supplemental Figure 8. Median Lp(a) levels and CV-events across studies utilizing latex-based assay

Abbreviation: MI: myocardial infarction


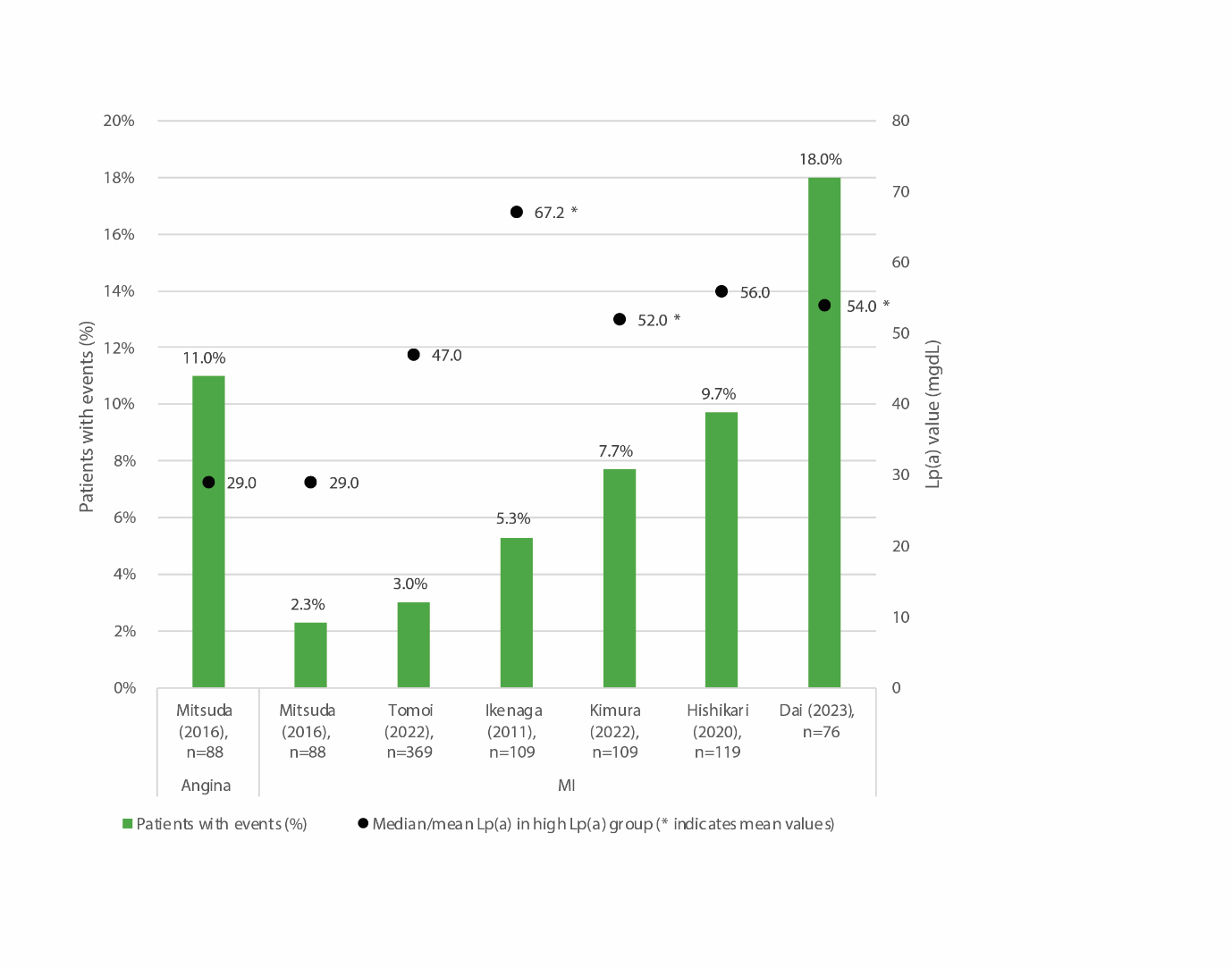


Supplemental Figure 9. Median Lp(a) levels and CV-events across all Lp(a) measurement kits

Abbreviation: MI: myocardial infarction


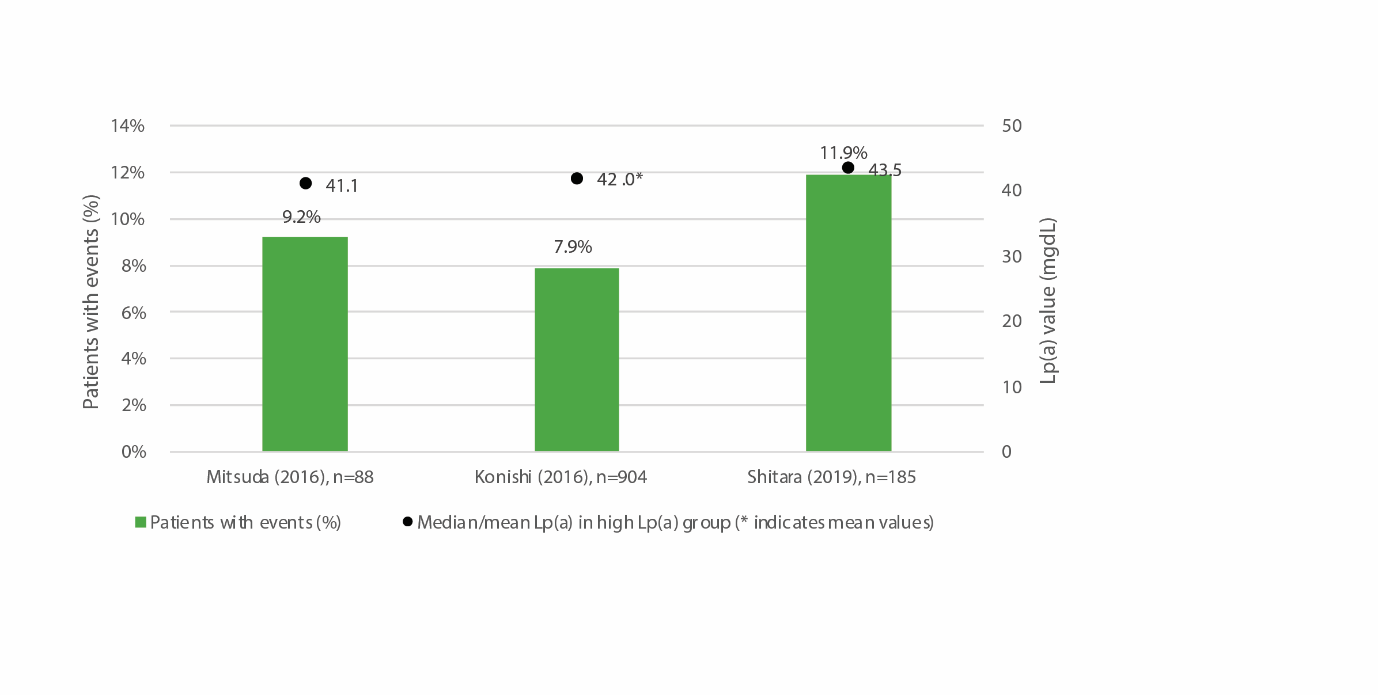
Supplemental Figure 10. Median/mean Lp(a) levels and ACS across studies utilizing latex-based assay

Abbreviation: ACS: acute coronary syndrome


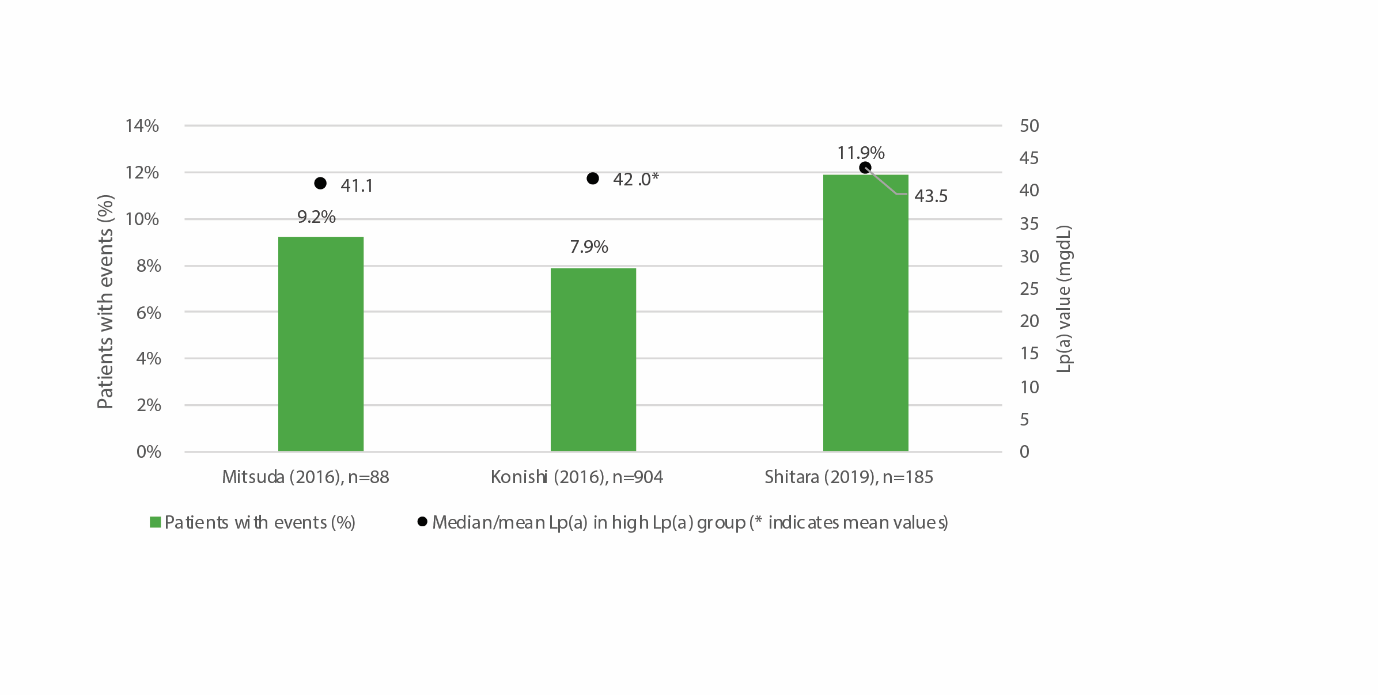


Supplemental Figure 11. Median/mean Lp(a) levels and ACS across all Lp(a) measurement kits

The studies reporting ACS in high Lp(a) utilized latex-based; therefore, the overall results remain unchanged.

Abbreviation: ACS: acute coronary syndrome
